# Phosphate uptake restriction, phosphate export, and polyphosphate synthesis contribute synergistically to cellular proliferation and survival

**DOI:** 10.1016/j.jbc.2023.105454

**Published:** 2023-11-08

**Authors:** Masahiro Takado, Tochi Komamura, Tomoki Nishimura, Ikkei Ohkubo, Keita Ohuchi, Tomohiro Matsumoto, Kojiro Takeda

**Affiliations:** 1 Radiation Biology Center, Graduate School of Biostudies, Kyoto University, Kyoto, Japan; 2 Graduate School of Natural Science, Konan University, Kobe, Japan; 3 Institute of Integrative Neurobiology, Konan University, Kobe, Japan

**Keywords:** phosphate, phosphate export, polyphosphate, the SPX domain, IBGC (idiopathetic basal ganglia calcification)

## Abstract

Phosphate (Pi) is a macronutrient, and Pi homeostasis is essential for life. Pi homeostasis has been intensively studied; however, many questions remain, even at the cellular level. Using *Schizosaccharomyces pombe*, we sought to better understand cellular Pi homeostasis and showed that three Pi regulators with SPX domains, Xpr1/Spx2, Pqr1, and the VTC complex synergistically contribute to Pi homeostasis to support cell proliferation and survival. SPX domains bind to inositol pyrophosphate and modulate activities of Pi-related proteins. Xpr1 is a plasma membrane protein and its Pi-exporting activity has been demonstrated in metazoan orthologs, but not in fungi. We first found that *S. pombe* Xpr1 is a Pi exporter, activity of which is regulated and accelerated in the mutants of Pqr1 and the VTC complex. Pqr1 is the ubiquitin ligase downregulating the Pi importers, Pho84 and Pho842. The VTC complex synthesizes polyphosphate in vacuoles. Triple deletion of Xpr1, Pqr1, and Vtc4, the catalytic core of the VTC complex, was nearly lethal in normal medium but survivable at lower [Pi]. All double-deletion mutants of the three genes were viable at normal Pi, but *Δpqr1Δxpr1* showed severe viability loss at high [Pi], accompanied by hyper-elevation of cellular total Pi and free Pi. This study suggests that the three cellular processes, restriction of Pi uptake, Pi export, and polyP synthesis, contribute synergistically to cell proliferation through maintenance of Pi homeostasis, leading to the hypothesis that cooperation between Pqr1, Xpr1, and the VTC complex protects the cytoplasm and/or the nucleus from lethal elevation of free Pi.

Phosphorus is the sixth most abundant element in living organisms and exists in the form of phosphate (PO_4_, hereafter Pi). Being central to energy metabolisms, a constituent of nucleic acids and lipid membranes, and serving various functions in intracellular signal transduction, Pi is involved in huge numbers of cellular biochemical reactions. Therefore, Pi homeostasis is absolutely pivotal in basic biology, agriculture, and medical sciences (1). In mammals, Pi is incorporated into the body from food, stored as calcium phosphate in bones, and excreted in urine by the kidneys, and Pi homeostasis at the organismal level is governed and fine-tuned by endocrine systems, for example, the FGF23-α-*klotho* system (1, 2, 3, 4). Loss of function mutants of α*-klotho* and FGF23 compromise phosphate metabolism (5, 6), resulting in pleiotropic phenotypes related to early senescence in mice (7). Hyperphosphatemia, a syndrome involving elevated serum [Pi], causes poor prognosis in chronic kidney disease patients. Thus Pi homeostasis at the organismal level has been intensively studied and is becoming well understood. Pi homeostasis in cells is also important, and its failure leads to diseases like primary familial brain calcification (PFBC) or idiopathic basal ganglia calcification, an abnormal accumulation of calcium phosphate in basal ganglia and other parts of the brain (8). As responsible genes for PFBC, two factors related to Pi homeostasis have been identified: *SLC20A2*, encoding the Pi transporters, Pit-2 and *XPR1* (xenotropic and polytropic murine leukemia retrovirus receptor)*,* encoding the SPX-EXS protein, the Pi exporter described later (8, 9, 10). Despite recent progress in understanding Pi homeostasis in mammals, as described above, many regulatory mechanisms remain unknown (1).

Intensive studies have been carried out on cellular Pi homeostasis by exploiting genetics of the budding yeast, *Saccharomyces cerevisiae*, and revealed the phosphatase (PHO) pathway (11, 12, 13). The PHO pathway is a transcriptional regulation system controlling the so-called PHO regulon, which consists of Pi-responsive genes, including Pho5, an acid phosphatase that digests organic phosphates in the environment, and Pho84, a major Pi transporter. By activating the PHO regulon, budding yeast cells are able to utilize extracellular Pi and to adapt to Pi-limiting conditions. Changes in cellular Pi concentration are transduced to the nucleus through the PHO pathway (14), the core of which is the Pho80/Pho85, cyclin/CDK complex, and its CDK inhibitor Pho81. Pho80/Pho85 phosphorylates and inactivates Pho4, the key transcription factor of the PHO pathway (15, 16, 17). At low [Pi], Pho81 inhibits Pho80/Pho85 but not at high [Pi]. The metabolic enzymes for inositol pyrophosphates (PP-IPs), for example, Kcs1, are required for proper PHO regulation (18) and binding state of inositol pyrophosphates like IP_7_ to Pho81 modulates its CDK-inhibiting activity (19, 20, 21). It was reported that IP_7_ binds to the C-terminal S1 region of Pho81 (22). The abundance of PP-IPs presumably fluctuates in response to available Pi concentration; therefore, the PHO pathway transduces information about Pi concentration *via* PP-IPs. Pho81 possesses an SPX (Syg1-Pho81-Xpr1) domain, found in many regulators of Pi metabolism. Later, the SPX domain was found to act as a binding site for PP-IP (23). Therefore, the PP-IP–related signals could be mediated *via* the SPX domains and others and orchestrate cellular responses to the perturbation of Pi environment. *S. cerevisiae* possesses ten SPX proteins and nine of them, Pho81, Vtc2, Vtc3, Vtc4, Vtc5, Gde1, Pho87, Pho90, and Pho91, are reportedly involved in Pi metabolism (Table 1) (21, 24). The one exception is Syg1, an SPX-EXS (Erd1-Xpr1-Syg1) protein occurring at the plasma membrane, which has not been investigated in the context of Pi regulation. Syg1 was originally identified as a genetic suppressor of mutations of G protein α subunit (25). Given its sharing domain structure, SPX-EXS, with human XPR1, the Pi exporter, *S. cerevisiae* Syg1 might be a potential Pi exporter too, but no concrete evidence has been reported. These SPX proteins may be regulated by PP-IPs in a coordinated fashion to achieve Pi homeostasis. In *S. cerevisiae*, regulatory mechanisms for Pi homeostasis have been well studied. However, not all core regulatory elements, such as Pho81, are conserved among eukaryotes, even in the kingdom of fungi.

**Table 1:** Comparison of SPX proteins from *Schizosaccharomyces pombe*, *Saccharomyces cerevisiae*, *Arabidopsis thaliana*, and *Homo sapiens*

*S. pombe*	*S. cerevisiae*	*A. thaliana*	*H. sapiens*	Molecular function
Vtc2 Vtc4	Vtc2/Phm1^b^ Vtc3/Phm2 Vtc4/Phm3	-	-	The VTC complex subunit polyP synthesis
-	Vtc5	-	-	polyP synthesis activator
-	Pho81	-	-	CDK (Pho80/Pho85) inhibitor
Pqr1/Spx1^a^	-	NLA NLA2	-	Pi quantity restriction RING-type ubiquitin ligasedownregulation of Pi transporter
Plt1	Pho87 Pho90 Pho91	SPX-MFS1-3	-	Pi transporter
Gde1	Gde1	-	-	Glycerophosphoryl diester phosphodiesterase
Xpr1/Spx2^a^	Syg1	PHO1-10	XPR1	Pi exporter, EXS domain
-	-	SPX1-4	-	containing solely SPX domain involved in the expression of phosphate-related genes

Hyphens indicate that an ortholog is not found in the genome.

a Spx1 and Spx2 are synonyms for Pqr1 and Xpr1, respectively (47).

b Phm1∼3 are synonyms for Vtc2∼4 (43).

In fission yeast, *Schizosaccharomyces pombe*, Pi-regulating genes, such as *pho1*^*+*^ (acid phosphatase), *pho84*^*+*^ (major Pi transporter), and *tgp1*^*+*^ (glycerophosphate transporter) constitute the PHO regulon and are transcriptionally regulated in response to Pi concentration (26, 27, 28, 29). Regulation of the PHO regulon in *S. pombe* has also been intensively investigated relatively recently, revealing involvement of phosphorylation states of C-terminal domain repeats of RNA polymerase II and long non-coding RNAs (29, 30, 31, 32). In *S. pombe,* too, changes in Pi concentration are likely mediated by PP-IPs *via* transcriptional regulation, as in *S. cerevisiae*. Six genes encoding SPX proteins are found in the genome and they may be involved in Pi homeostasis (Table 1). Although it sounds analogous to the PHO pathway of *S. cerevisiae*, *S. pombe* lacks either SPX-CDK inhibitor, Pho81, pivotal for the PHO pathway or the functional ortholog of CDK, Pho85, which is similar to Pef1 in *S. pombe.* Pef1 is involved in meiosis progression and chromosomal cohesion rather than in the regulation of Pi homeostasis (33, 34, 35). Regulatory mechanisms for Pi homeostasis in *S. pombe*, many parts of which are still unclear, may differ considerably from those in *S. cerevisiae*.

In our previous study, we reported that the fission yeast Pqr1, a RING-type ubiquitin ligase with an SPX domain (SPX-RING), contributes to Pi homeostasis, restricting Pi uptake by downregulating the major Pi transporters, Pho84 and Pho842 (36). Loss of Pqr1 results in excessive influx of Pi into the cell, causing failure of phosphate quantity restriction. Consequently, it evokes hyper-accumulation of inorganic polyphosphate (polyP), a Pi polymer, synthesized by the VTC complex in vacuolar membranes. Hyper-accumulation of polyP in vacuolar lumens interferes with proper proteolysis in the vacuoles, which is indispensable for autophagic recycling of amino acids, thereby causing cell death during cellular quiescence (G_0_ phase) induced by nitrogen-limitation (36, 37, 38, 39). In Pqr1-deficient cells, inactivation of the VTC complex diminishes polyP and restores proteolytic activity in vacuoles. Interestingly, Pqr1, the key factor in Pi homeostasis, has no counterpart in *S. cerevisiae* but is functionally homologous to *NLA,* an SPX-RING ubiquitin ligase central to Pi homeostasis in *Arabidopsis thaliana* (40, 41, 42). The VTC complex is the polyP synthase identified in *S. cerevisiae* and comprises Vtc1, Vtc2, and catalytic Vtc4, or Vtc1, Vtc3, and Vtc4 (43, 44). As Vtc2, Vtc3, Vtc4, and an additional activator, Vtc5, possess an SPX domain in the N terminus, the VTC complex is also under the influence of PP-IPs (24, 45). Although the subunit composition is slightly different from that of *S. cerevisiae*, the VTC complex in *S. pombe*, composed of Vtc1, Vtc2, and Vtc4, is responsible for synthesizing cellular polyP too (36, 46, 47). While sharing common features, the regulatory mechanisms of Pi homeostasis in *S. pombe* differ from those of *S. cerevisiae*. Identifying mechanisms of Pi homeostasis in *S. pombe* will provide another standard and useful information to understand Pi homeostasis in eukaryotes.

The present study used Pqr1 as a starting point and attempted to further understanding of Pi regulation using *S. pombe* as a model. As described above, total Pi in the cell increases 3∼5-fold by inactivating Pqr1 (36). In this study, we first discovered that in Pqr1-deficient cells, a hyper-increase in total Pi is suppressed by deletion of Vtc4, a catalytic subunit of the VTC complex, so we investigated underlying molecular mechanisms. As a possible explanation for the suppression of hyper-accumulation of Pi, the Pi export process was considered and a potential Pi exporter encoded by SPCC1827.07c was investigated. Because our subsequent analysis revealed that it is a functional ortholog of human Xpr1, a known Pi exporter, we designated SPCC1827.07c as *xpr1*^*+*^. During preparation of this manuscript, we found that Drs. Beate Schwer, Stewart Shuman and colleagues named this gene *spx2*^*+*^ (47); however, in this manuscript, we have adopted the name, *xpr1*^*+*^. We show that *S. pombe* has Xpr1-dependent Pi export activity and contributes to normal proliferation and cellular viability, by maintaining Pi homeostasis synergistically in combination with Pqr1 and the VTC complex, leading to the hypothesis that the trio of SPX factors, Pqr1, Xpr1, and the VTC complex, protects the cytoplasm and/or the nucleus from lethal elevation of free Pi.

## Results

### Hyperaccumulation of cellular Pi in Δpqr1 is suppressed by inactivation of the VTC complex

**Figure 1:**
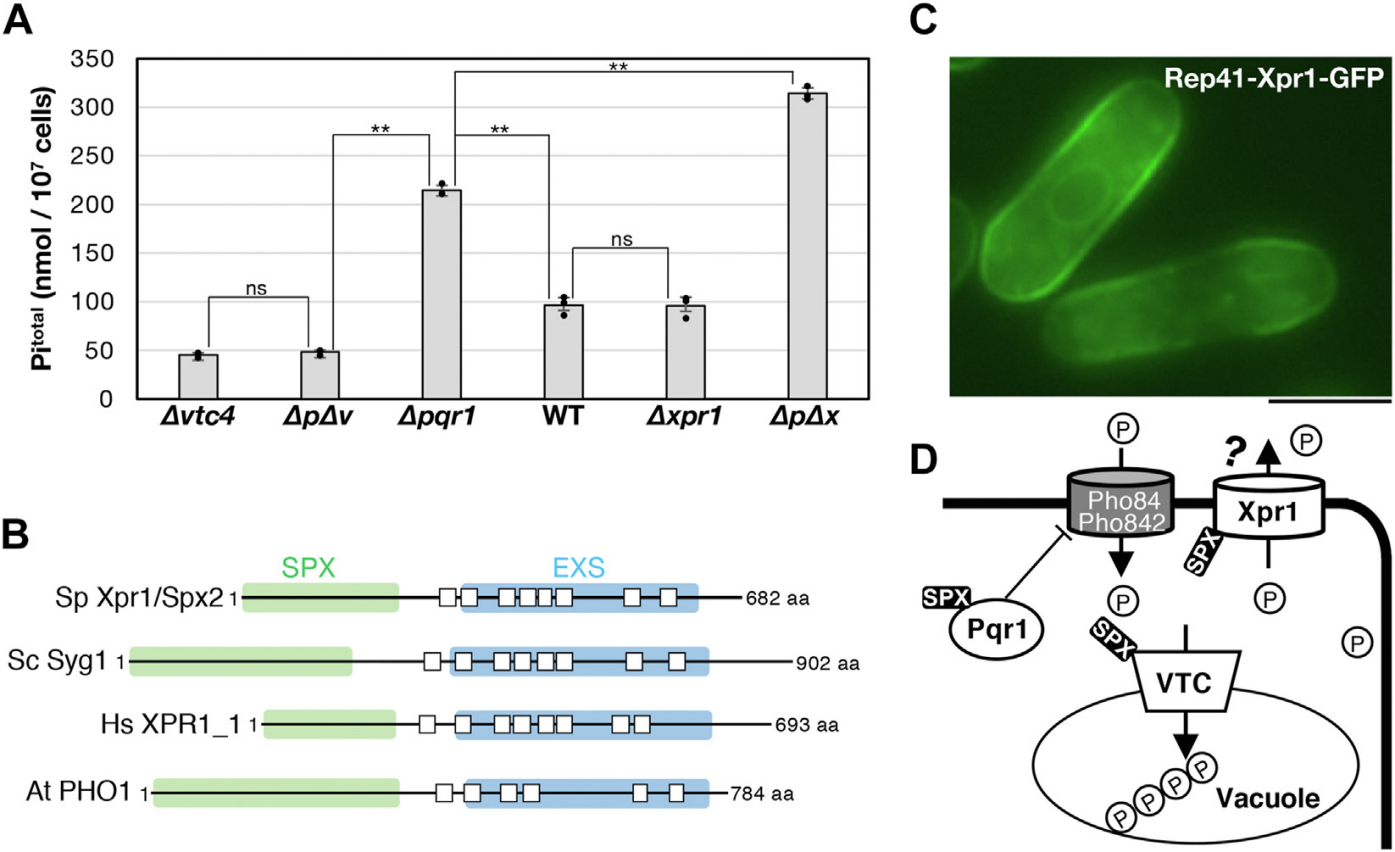
***Schizosaccharomyces pombe* Xpr1, a potential Pi exporter, is involved in Pi homeostasis.***A*, total intracellular Pi (Pi^total^) was measured in indicated strains cultured in normal synthetic medium EMM2 (Pi 15 mM). Experiments were repeated 3×, and individual data points, means, and SDs are presented. A double asterisk (∗∗) or 'ns' means the difference is or is not statistically significant by Student's *t* test (*p* < 0.02), respectively. *ΔpΔv* and *ΔpΔx* indicate *Δpqr1Δvtc4* and *Δpqr1Δxpr1*, respectively. *B*, domain structures of Xpr1 orthologs. *White squares* represent estimated transmembrane regions. The N-terminal SPX domain and the C-terminal EXS domain are shown in *green* and *blue*, respectively. Sp, *Schizosaccharomyces pombe*; Sc, *Saccharomyces cerevisiae*; Hs, *Homo sapiens*; At, *Arabidopsis thaliana*. *C*, intracellular localization of Xpr1-GFP expressed from multicopy plasmids with the inducible nmt41 promoter. Bar represents 5 μm. *D*, the three SPX factors, Pqr1, VTC, and Xpr1 in Pi homeostasis in *S. pombe*.

Our previous study suggested that the SPX-RING-type ubiquitin ligase, Pqr1 restricts Pi uptake by downregulating Pho84 and Pho842, major high-affinity Pi transporters in *S. pombe* (36). In cells lacking Pqr1, Pi uptake may be enhanced, resulting in hyper-accumulation of polyP in vacuolar lumens, which interferes with proper vacuolar proteolysis in the last step of autophagy. When the polyP synthase, the VTC complex, is inactivated in *Δpqr1* (a *pqr1*^*+*^ gene deletion strain), abnormal accumulation of polyP was suppressed. This result led us to question how cells manage excess Pi uptake without synthesizing polyP in vacuoles. To address this, total amounts of Pi in cells (Pi^total^) were quantified and compared among the following: WT, *Δpqr1*, *Δvtc4,* and *Δpqr1Δvtc4* (Fig. 1*A*). In *Δpqr1*, Pi^total^ was 214.5 ± 5.1 nmol/10^7^ cells, while 96.7 ± 7.6 in WT, consistent with our previous study (36). Interestingly, the hyper-accumulation of Pi in *Δpqr1* was suppressed in *Δpqr1Δvtc4* cells, which are not able to synthesize polyP. Pi^total^ in *Δpqr1Δvtc4* was around 48.3 ± 2.1 nmol/10^7^ cells, significantly reduced compared to *Δpqr1* (*p* < 0.02). The Pi^total^ of *Δvtc4* was 45.6 ± 2.0 nmol/10^7^ cells, virtually the same as that of *Δpqr1Δvtc4* (nonsignificant difference, *p* > 0.2). These results suggested that either the majority of over-incorporated Pi in *Δpqr1* is converted to polyP *via* the VTC complex or that *S. pombe* may possess alternative mechanisms to reduce Pi^total^ other than restricting Pi uptake by Pqr1. Such mechanisms may be keys to cellular Pi homeostasis and its molecular basis would be machinery to export excess Pi from the cell or a Pqr1-independent inhibitor for Pi uptake. In this study, we focused on the first possibility, the machinery needed to export Pi.

### Gene deletion of a potential Pi exporter, Xpr1, affects cellular Pi amount

Previous studies have reported that a plasma membrane protein, Xpr1, promotes Pi export from mammalian cells (9). Xpr1 possesses an SPX domain at its N terminus and an EXS domain at its C terminus (Fig. 1*B*). The SPX domain reportedly functions as a Pi sensor and to bind signaling molecules, PP-IPs, like IP_7_ and IP_8_ (21). The EXS domain is composed of eight transmembrane regions. Orthologs of Xpr1, SPX-EXS proteins, are found in all metazoans (Table 1). *S. cerevisiae* Syg1, orthologous to Xpr1, has not been functionally analyzed in the context of Pi homeostasis. *S. pombe* has one gene, SPCC1827.07c encoding an SPX-EXS protein, function of which also has not been thoroughly investigated. We designated SPCC1827.07c as *xpr1*^*+*^ and examined its roles in Pi homeostasis in *S. pombe*.

Then we compared Pi^total^ in WT, *Δpqr1,* and *Δxpr1* (Fig. 1*A*) and unexpectedly found that Pi^total^ in *Δxpr1* was 95.8 ± 8.9 nmol/10^7^ cells, similar to that in WT. Then, a *Δpqr1Δxpr1* double-deletion mutant was created and its Pi^total^ was 314.1 ± 5.7 nmol/10^7^ cells, approximately a three-fold increase from WT. Importantly, Pi^total^ in *Δpqr1Δxpr1* was also significantly higher than that of *Δpqr1* (214.5 ± 5.1 nmol/10^7^ cells, *p* < 0.02). Therefore, Pqr1 and Xpr1 may synergistically restrict Pi^total^, suggesting that these two are located in distinct pathways, consistent with the idea that Xpr1 is a Pi exporter. A *Δxpr1Δvtc4* double-deletion mutant was also created and its Pi^total^ was similar to that of *Δvtc4* (Fig. S1).

The intracellular localization of Xpr1 was examined by expressing Xpr1-GFP from multicopy plasmids with the inducible promoter, *nmt41.* It was localized mainly in the cell periphery, probably at the plasma membrane (Fig. 1*C*), consistent with its role in Pi export. Taken together, these results suggest that Xpr1/Spx2, a potential Pi exporter, may contribute to cellular Pi homeostasis with Pqr1 and the VTC complex (Fig. 1*D*).

### The synthetic growth defect of Δpqr1Δxpr1Δvtc4

**Figure 2:**
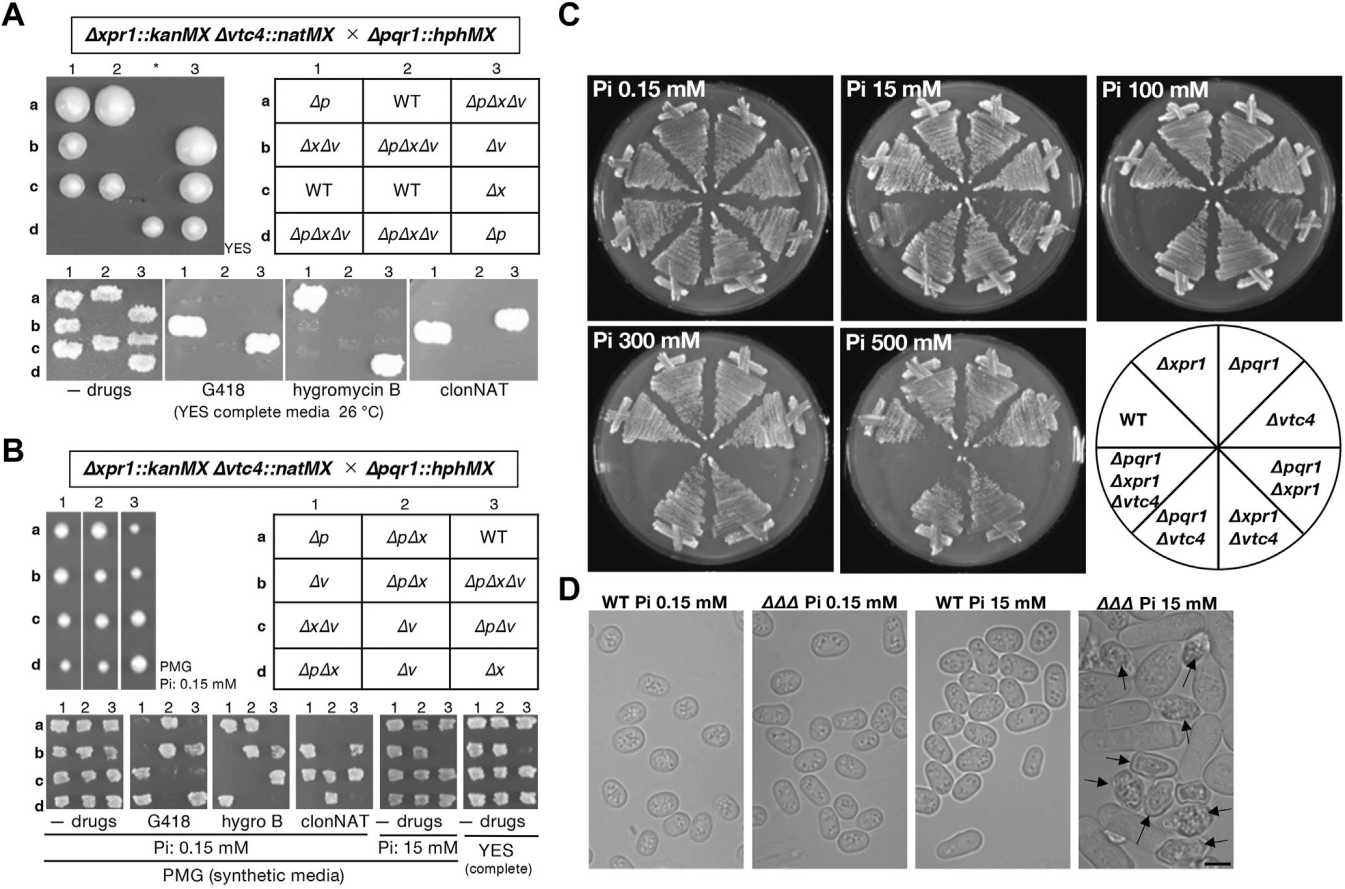
***Δpqr1Δxpr1Δvtc4* and *Δpqr1Δxpr1* are hyper-sensitive to higher Pi concentration.***A*, the triple deletion of *pqr1*^*+*^, *xpr1*^*+*^, and *vtc4*^*+*^ is synthetic lethal on complete YES medium. The double mutant *Δxpr1Δvtc4* was mated with *Δpqr1* and four spores per ascus were dissected on an YES plate (tetrad dissection, *upper left*). An asterisk indicates a dissection mistake. Each gene of *pqr1*^+^, *xpr1*^*+*^, or *vtc4*^*+*^ was substituted with a conventional drug-resistant marker gene: *hphMX* for hygromycin B, *kanMX* for G418, and *natMX* for clonNAT. By checking the drug sensitivity of each colony (*bottom*), genotypes of dissected spores were analyzed (*upper right*). *Δp*, *Δx*, and *Δv* indicate *Δpqr1*, *Δxpr1*, and *Δvtc4*, respectively. *B*, *Δpqr1Δxpr1Δvtc4* is viable on low Pi medium. As in *A*, tetrad dissection was done on PMG0.15 mM Pi instead. The *ΔpΔxΔv* (3b) was resistant to all three drugs and did not form colonies on PMG^15mM Pi^ or YES, a complete medium. *C*, proliferation of indicated strains was examined on EMM2 plates with various concentrations of Pi (0.15–500 mM). Normal EMM2 contains 15 mM Pi. *D*, *Δpqr1Δxpr1Δvtc4* cells (ΔΔΔ in the figure) showed abnormal morphology on EMM2 with 15 mM Pi. Cells from agar plates were observed by microscope. Arrowheads indicate collapsed cells. Bar represents 5 μm.

To confirm the involvement of Xpr1 in Pi homeostasis, we created a triple gene-deletion mutant of *pqr1*^*+*^, *xpr1*^*+*^, and *vtc4*^*+*^ (*Δpqr1Δxpr1Δvtc4*). To this end, the *Δxpr1Δvtc4* strain was mated with *Δpqr1* and yeast tetrad analysis was carried out (Fig. 2*A*). Asci (three examples shown in Figure 2*A* upper left) were dissected to four spores (a ∼ d) on an YES (complete medium) plate and incubated at 26 °C for several days. Spores germinated and formed colonies, and genotypes were subsequently analyzed (Fig. 2*A* upper right and bottom). We found that spores assumed to be *Δpqr1Δxpr1Δvtc4* did not form colonies, suggesting synthetic lethality of gene deletions of *pqr1*^*+*^, *xpr1*^*+*^, and *vtc4*^*+*^ on YES. We considered the possibility that germination or growth of *Δpqr1Δxpr1Δvtc4* is affected by Pi contained in YES. Then we quantified free Pi (orthophosphate) and total Pi (including organophosphorus compounds like phytate) in YES (Fig. S2) and found that concentrations of free Pi and total Pi in YES were 1.1 and 1.3 mM, respectively. Therefore, we next performed the same tetrad analysis on the lower Pi medium plate PMG (modified PMG synthetic minimal medium containing 0.15 mM Pi) (Fig. 2*B*). Normal PMG contains 15 mM Pi, as does normal EMM2 synthetic minimal medium (48). This time, we obtained *Δpqr1Δxpr1Δvtc4* colonies on PMG with 0.15 mM Pi, which did not grow on either normal PMG (15 mM Pi) or YES. We concluded that *Δpqr1Δxpr1Δvtc4* shows a synthetic severe growth defect at the normal [Pi] usually used in *S. pombe* studies.

### Pqr1, Xpr1, and Vtc4 synergistically contribute to high Pi tolerance

To clarify how the three SPX factors, Pqr1, Xpr1, and the VTC complex, help sustain cellular proliferation through maintenance of Pi homeostasis, we examined the effect of [Pi] in the medium on cell growth of all possible gene deletion mutants of the three genes (Fig. 2*C*). All tested strains were grown in 0.15 mM Pi. Growth of *Δpqr1Δxpr1Δvtc4* was severely inhibited at 15 mM Pi and was completely blocked at 100 mM Pi or more. An aliquot of cells of *Δpqr1Δxpr1Δvtc4* was taken from the agar plates and observed microscopically. Cells were severely deformed/swollen and many were collapsed and probably dying (Figs. 2*D* and S2). These abnormal phenotypes of *Δpqr1Δxpr1Δvtc4* were dependent on [Pi] and were only seen at 15 mM Pi and never at 0.15 mM Pi. Notably, *Δpqr1Δxpr1Δvtc4* can form colonies at 0.15 mM Pi but is genetically unstable, probably due to accumulation of suppressor mutations. For this reason, we did not investigate this triple mutant further in this study.

All double mutants, *Δpqr1Δxpr1*, *Δpqr1Δvtc4,* and *Δxpr1Δvtc4*, were able to form colonies at 100 mM Pi. *Δpqr1Δxpr1* showed slow growth at 100 mM Pi and failed to grow at 300 mM Pi (Fig. 2*C*). Contrarily, *Δpqr1Δvtc4* and *Δxpr1Δvtc4* grew as well as WT and single mutants even grew at 500 mM Pi. Cells of the three double mutants were observed at 0.15 or 15 mM and show no collapsed or dead cells (Fig. S3).

These results suggest that the three SPX factors, Pqr1, Xpr1, and the VTC complex, contribute synergistically to sustain vegetative proliferation under normal and high Pi conditions. Considering that only *Δpqr1Δxpr1Δvtc4* showed severe defects at normal [Pi] (15 mM), while all double mutants were rather similar to WT at 15 mM Pi, molecular functions of Pqr1, Xpr1, and the VTC complex are likely to be essential in the absence of the other two.

### Loss of both Pqr1 and Xpr1 causes hyper-elevation of intracellular Pi levels

**Figure 3:**
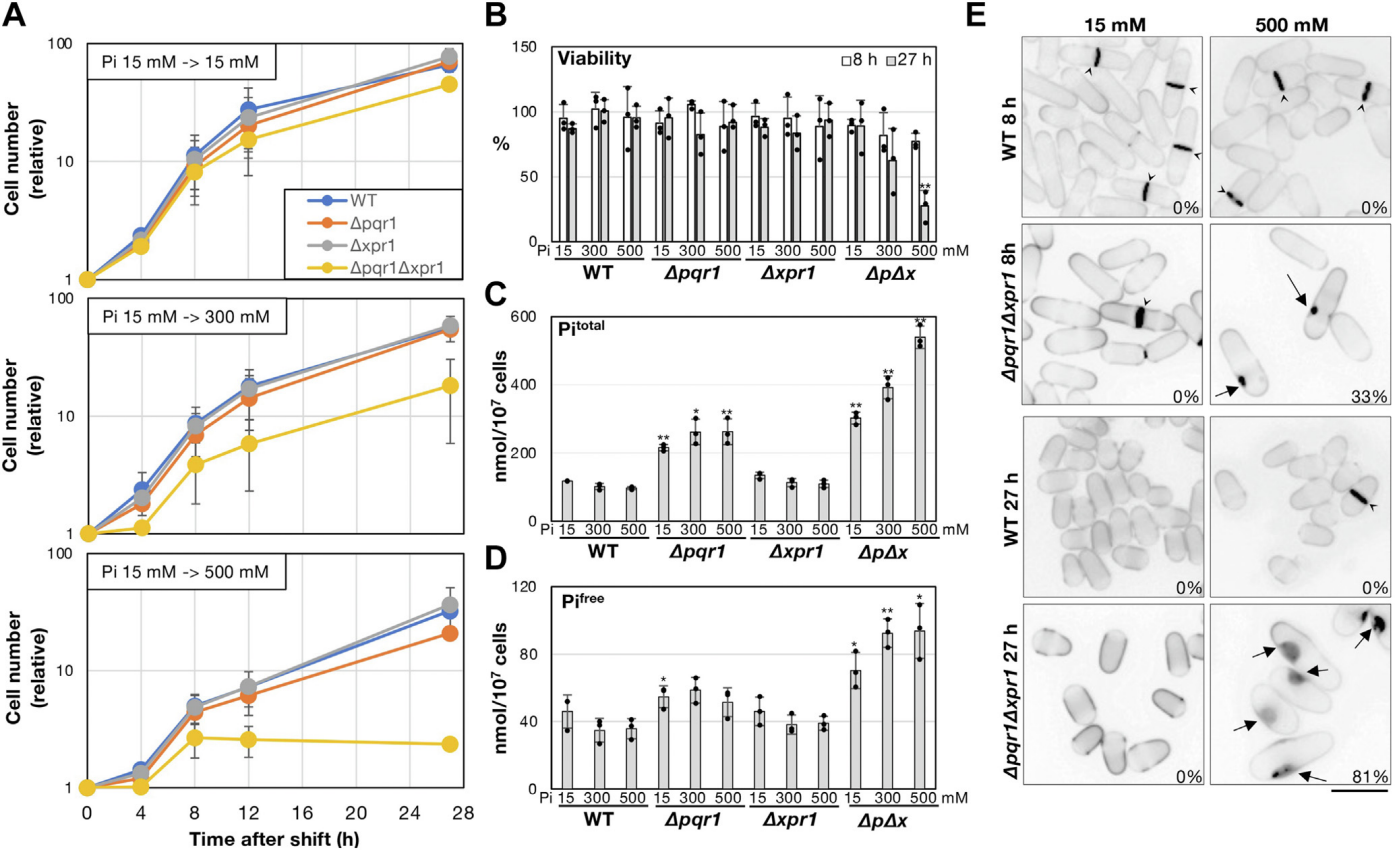
***Δpqr1Δxpr1* showed hyper-accumulation of Pi and lost viability under higher Pi.***A*, growth curves of indicated strains after the shift to 15, 300, or 500 mM Pi EMM2 from normal EMM2 (Pi, 15 mM). Y-axes indicate cell concentrations in the media measured with a particle counter and relative values are plotted (setting value at 0 h to 1.0). *B*, cellular viabilities at 8 and 27 h after medium shift. *ΔpΔx* indicates *Δpqr1Δxpr1*. *C*, total amounts of intracellular Pi (Pi^total^) of indicated strains at 8 h after medium shift. Presented data were normalized by cell numbers (nmol Pi/10^7^ cells). *D*, amounts of intracellular free Pi at 8 h. For B-D, experiments were repeated three times, and individual data points, means, and SDs were presented. Data of WT and each mutant were statistically analyzed: an asterisk (∗) for significantly different (*p* < 0.05) and a double asterisk (∗∗) for *p* < 0.02. Others were not significantly different from corresponding WT results. *E*, cellular morphologies were examined with Calcofluor White (CW) to stain cell walls. Structures indicated by arrowheads are septa formed before cytokinesis. Arrows indicate abnormal invaginations observed in *Δpqr1Δxpr1*. Materials stained strongly with CW were deposited in the invaginated space. The percentage of cells with invaginations is shown. Bar represents 5 μm.

In Figure 2, we found that *Δpqr1Δxpr1* showed the most severe growth defect, next to *Δpqr1Δxpr1Δvtc4*, in the higher [Pi]. As the triple deletion mutant *Δpqr1Δxpr1Δvtc4* was difficult to investigate, we focused on investigating *Δpqr1Δxpr1*. To address whether a failure in Pi homeostasis is caused in *Δpqr1Δxpr1*, we performed a time-course analysis of cell growth, viability, [Pi], and cell morphology of *Δpqr1Δxpr1*, *Δpqr1,* and *Δxpr1* at 15, 300, and 500 mM Pi (Fig. 3). First, time-course analyses showed that proliferation of *Δpqr1Δxpr1* was inhibited at 300 and 500 mM Pi (Fig. 3*A*). At 27 h after the shift to 500 mM Pi, viability of *Δpqr1Δxpr1* had decreased to 27.4 ± 11.0%, while the other strains sustained significantly higher viabilities (*p* < 0.02): 95.3 ± 9.0%, 91.9 ± 13.7%, and 93.4 ± 13.0% in WT, *Δpqr1,* and *Δxpr1*, respectively. At either 15 or 300 mM Pi, no significant loss of viability was seen in *Δpqr1Δxpr1* and other strains (Fig. 3*B*). If the growth defect of *Δpqr1Δxpr1* is caused by defective regulation of Pi homeostasis, we supposed that Pi^total^ must be affected; therefore, we quantified Pi^total^ of these strains at 15, 300, and 500 mM Pi, 8 h after the medium shift (Fig. 3*C*). As already shown in Figure 1, *Δpqr1Δxpr1* showed extreme accumulation of Pi, and *Δpqr1* was the second at 15 mM Pi. In *Δpqr1Δxpr1*, values of Pi^total^ were 302.3 ± 17.4, 392.3 ± 33.1, and 539.6 ± 32.9 mM/10^7^ cells at 15, 300, and 500 mM Pi, respectively, increasing with medium Pi concentration. In sharp contrast, Pi^total^ of WT and *Δxpr1* did not increase at 300 and 500 mM Pi (Fig. 3*C*), suggesting that Pi homeostasis is sustained in these two strains, if Pqr1 is functional. In *Δpqr1*, values of Pi^total^ were 215.5 ± 9.3, 261.8 ± 37.4, and 262.4 ± 37.7 mM/10^7^ cells at 15, 300, and 500 mM Pi, respectively. These values were significantly higher than those of WT or *Δxpr1* (*p*-values shown in Fig. 3) and increased with medium Pi concentration, although the increase ratio was far smaller than in *Δpqr1Δxpr1*.

As Pi^total^ represents the sum of all forms of Pi in the cell (free Pi, nucleic acids, polyP, phospholipids, etc.), we next examined the level of intracellular free Pi (Pi^free^) in WT, *Δpqr1*, *Δxpr1,* and *Δpqr1Δxpr1* at 15, 300, and 500 mM Pi, 8 h after the medium shift. In *Δpqr1Δxpr1*, Pi^free^ were 70.3 ± 10.7, 92.5 ± 8.5, and 93.8 ± 16.3 nmol/10^7^ cells in 15, 300, and 500 mM Pi, respectively, significantly higher than in WT, 46.9 ± 9.9, 34.8 ± 7.0, and 35.8 ± 5.9 (*p*-values shown in Fig. 3). Pi^free^ of WT is sustained around 35∼47 nmol/10^7^ cells, regardless of medium [Pi], suggesting that this level of Pi^free^ may be homeostatically maintained since it is suitable for the physiology of *S. pombe*. Pi^free^ of *Δpqr1* tended to be higher than that of WT, although the difference was significant (*p* < 0.05) only at 15 mM Pi. The Pi^free^ of *Δxpr1* was similar to that of WT, as seen in Pi^total^, suggesting that the contribution of Xpr1 to Pi homeostasis may be minor in the presence of functional Pqr1 at extracellular [Pi] below 500 mM. This is consistent with Figure 3*B* showing no viability loss in *Δxpr1*. These results suggested that Pi homeostasis was compromised and free Pi hyper-accumulated in *Δpqr1Δxpr1* in higher Pi medium. Taken together, we conclude that Pqr1 and Xpr1 contribute synergistically to prevent lethal elevation of [Pi] in the cell so as to sustain proliferation.

### Abnormal morphology of Δpqr1Δxpr1 cells at higher Pi

What defects are caused by abnormal elevation of cellular Pi level? To answer this question, we examined cellular morphology by staining cell walls with Calcofluor White (CW) dye (Figs. 3*D* and S4). In *Δpqr1Δxpr1*, abnormal invagination of the plasma membrane was frequently observed at high Pi (0, 33, and 81% at 0, 8, and 27 h in 500 mM Pi, and 17% at 27 h in 300 mM Pi, respectively). In other strains, such invagination was not seen. In the invaginated space, probable cell wall materials were deposited, judging from CW staining, which detects polysaccharides. A similar phenotype was invoked by overexpression of Rga2, the Rho GAP, or by gene deletion of *klf1*^*+*^, a Krüppel-like transcription factor (49, 50).

### Xpr1-dependent Pi export in *S. pombe*

**Figure 4:**
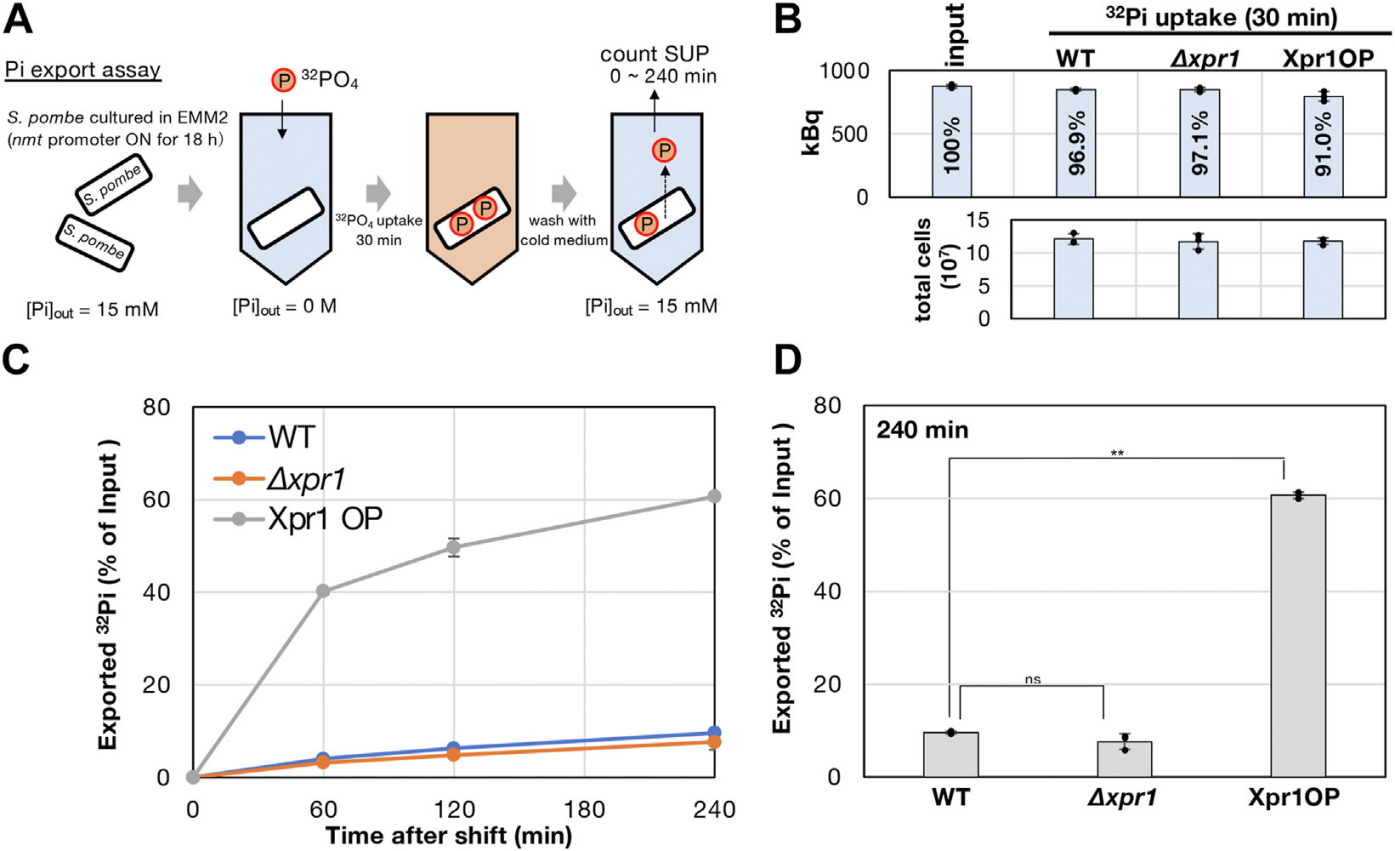
**Xpr1 overexpression accelerates Pi export.***A*, the scheme of Pi export assay. To induce gene expression by *nmt41* promoter, cells were cultured in EMM2 without vitamin B1 for 18 h at 26 °C. *B*, confirmation of ^32^Pi uptake. After incorporation of ^32^Pi into cells, aliquots of cells were taken and total amounts of ^32^Pi in cells were measured with a liquid scintillation counter. The amount of input ^32^Pi was also measured and compared with the incorporated ^32^Pi (*upper graph*). Percentages in the graph indicate ratios of ^32^Pi in the cells to the input. Cell numbers used are also shown (*bottom*). Experiments were repeated three times and individual data points, means, and SDs are shown. *C*, time-course analysis of Pi export in 15 mM Pi EMM2. The Y axis represents percentages of ^32^Pi exported from cells to incorporated total ^32^Pi. *D*, comparison of exported ^32^Pi at 240 min after the chase. The double asterisk indicates that the difference is statistically significant (∗∗ for *p* < 0.02). 'ns' means not significant.

Our results suggest that a potential Pi exporter, Xpr1, is important for Pi homeostasis in *S. pombe*. Is *S. pombe* Xpr1 a genuine Pi exporter? Budding yeast Syg1, orthologous to Xpr1, has not been investigated in the context of Pi metabolism (24, 25). Rather, to the best of our knowledge, Pi export itself has not been well investigated in fungi and no established protocol was available. Therefore, in this study, we developed a method to assay Pi export activity in *S. pombe* (Fig. 4*A*) and then we examined the activities of Pi export in *Δxpr1* transformed with an empty vector (*Δxpr1*) or with an Xpr1-GFP plasmid (Xpr1-OP), controlled with the inducible *nmt41* promoter (Fig. 4, *B*–*D* for Pi export assay). A strain (h^−^ *leu-32*) transformed with an empty vector was also used as a control (WT).

The cells in mid-log phase were washed and resuspended in EMM2-P (EMM2 without Pi), followed by the administration of KH_2_^32^PO_4_. We confirmed that ^32^Pi was efficiently incorporated in virtually the same manner in all tested strains (Fig. 4*B*). In the Pi export assay (Fig. 4, *C* and *D*), Xpr1-OP showed significantly stronger radioactivity in supernatant than *Δxpr1* (*p* < 0.02), showing Xpr1-dependent Pi export activity. The Pi export of WT was slightly, but reproducibly, stronger than *Δxpr1*. As shown in Fig. S5, Xpr1-GFP was indeed expressed. We detected two bands of Xpr1-GFP, implying that Xpr1 may be posttranslationally modified or partially cleaved, although we did not further investigate the nature of the modification. These results are consistent with the idea that *S. pombe* Xpr1 has Pi-exporting activity. Clearly, the radioactivity in the supernatant of *Δxpr1* was slowly but gradually increased (Fig. 4*C*), suggesting that Xpr1 is not a sole Pi exporter, but *S. pombe* has other cryptic—not yet identified—Pi exporter(s).

### The absence of Pqr1 or Vtc4 accelerates Pi export in an Xpr1-dependent manner

**Figure 5:**
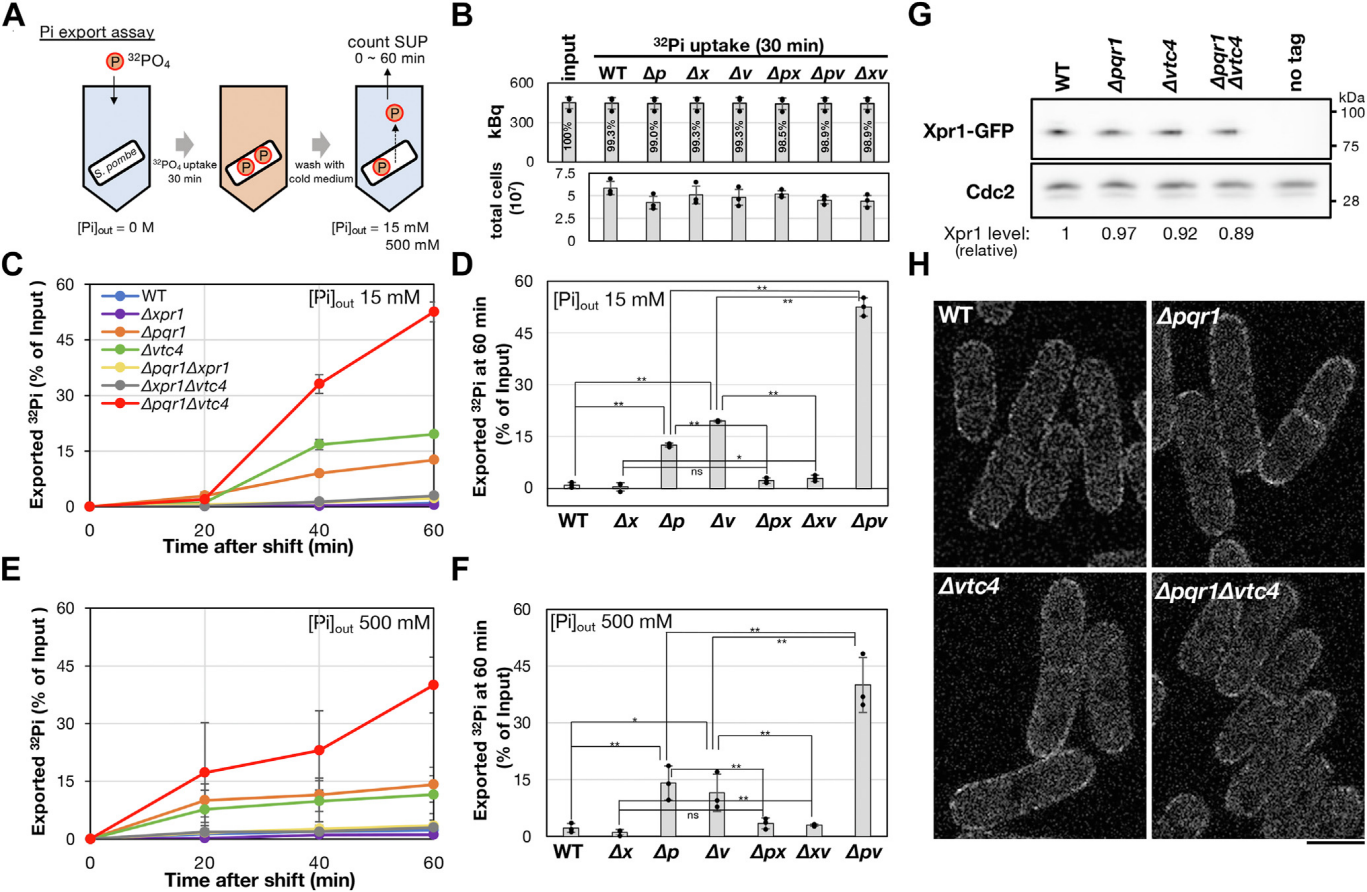
**Xpr1-dependent Pi export is accelerated in the absence of Pqr1 or Vtc4.***A*, the scheme of Pi export assay. *B*, confirmation of ^32^Pi uptake in tested strains. Experiments were repeated three times and individual data points, means, and SDs are shown. *C*, time-course analysis of Pi export in 15 mM Pi EMM2. The Y axis represents percentages of ^32^Pi exported from cells to incorporated total ^32^Pi. *D*, comparison of exported ^32^Pi in 15 mM Pi at 60 min after the chase. Single or double asterisks indicate that the difference is statistically significant (∗ for *p* < 0.05, ∗∗ for *p* < 0.02). 'ns' means not significant. *E*, time-course analysis similar to (*C*), instead the medium contained 500 mM Pi. *F*, similar to *D*, instead the medium contained 500 mM Pi. *G*, protein levels of Xpr1-GFP were examined by immunoblot and compared. The medium used for culture was EMM2 containing 15 mM Pi. Each signal intensity of Xpr1-GFP was normalized by Cdc2, the loading control, and the relative level of Xpr1-GFP is shown below the blot (the ratio of Xpr1-GFP level of each strain to that of WT). Xpr1-GFP levels were not increased by gene deletion of *pqr1*^*+*^ or *vtc4*^*+*^. *H*, localization of Xpr1-GFP expressed from the chromosomally integrated gene, controlled by its own promoter. Xpr1-GFP was enriched in the cell periphery in all tested strains. Bar represents 5 μm.

Next, we examined whether Xpr1-dependent Pi export is affected by the functions of other SPX proteins, Pqr1 and the VTC complex (Fig. 5). Similar to the previous assay, cells of indicated strains in mid-log phase were washed and resuspended in EMM2-P, followed by ^32^Pi administration (Fig. 5*A*). In this assay, Pi export was examined in EMM2 containing 15 or 500 mM Pi. First, we confirmed that ^32^Pi was efficiently incorporated in virtually the same manner in all tested strains (Fig. 5*B*).

In the Pi export assay, at 15 mM Pi, neither WT nor *Δxpr1* showed much radioactivity in the supernatant, as seen in Figure 4 (Fig. 5*C* for time-course, D for comparison at 60 min). However, *Δpqr1* and *Δvtc4* showed significantly stronger radioactivity in supernatants than WT (*p* < 0.02), depending on the presence of Xpr1. Strong radioactivity was not seen in the supernatant of either *Δpqr1Δxpr1* or *Δxpr1Δvtc4*. These results suggest that *S. pombe* has Xpr1-dependent Pi export activity; Xpr1-dependent Pi export is accelerated by either *Δpqr1* or *Δvtc4*. Importantly, the elevation of Pi export activity in *Δpqr1* and *Δvtc4* is synergistic. In *Δpqr1Δvtc4*, exported Pi was far greater than that in the single mutant (Fig. 5*D* for statistical analyses). Similarly, we examined Pi export at 500 mM Pi (Fig. 5, *E* and *F*) and obtained results similar to those at 15 mM Pi. The Pi export activity of WT at 500 mM Pi was similar to that at 15 mM. As both Pi^total^ and Pi^free^ were homeostatically sustained in WT and *Δxpr1* at 15 ∼ 500 mM Pi (Fig. 3, *C* and *D*), necessity of Xpr1-dependent Pi export might be limited in the tested conditions. The timing of activation of Pi export in *Δpqr1*, *Δvtc4,* and *Δpqr1Δvtc4* was earlier at 500 mM Pi than at 15 mM Pi (Fig. 5, *C* and *E*). Additionally, we noticed that Pi export activities in *Δpqr1Δxpr1* and *Δxpr1Δvtc4* were slightly but reproducibly higher than in *Δxpr1* at either 15 mM or 500 mM Pi (Fig. 5, *D* and *F*), suggesting that *S. pombe* may possess Pi exporters other than Xpr1. Thus, taken the results of Figures 4 and 5 together, we conclude that *S. pombe* has Xpr1-dependent Pi export activity, which is regulatable and activated by the absence of either Pqr1 or the VTC complex or both.

### Protein level and cell peripheral localization of Xpr1 with deletion of pqr1^+^, vtc4^+,^ or both

The Pi export assay shown in Figure 5 suggested that Xpr1-dependent Pi export activity is elevated in the absence of Pqr1 or the VTC complex. To obtain mechanistic insight into how Xpr1 is regulated, we examined the protein level of Xpr1 and its intracellular localization in WT, *Δpqr1*, *Δvtc4,* and *Δpqr1Δvtc4* cells. To this end, a GFP gene was C-terminally fused to the endogenous *xpr1*^*+*^ gene (chromosomally integrated). Transcription of the fusion gene was regulated by the *xpr1*^*+*^ promoter. We found that Xpr1-GFP did not increase in *Δpqr1*, *Δvtc4,* or *Δpqr1Δvtc4* (Fig. 5*G*). Accelerated Pi export in these mutants may not be caused by increased amounts of Xpr1 protein. Next, we investigated localization of Xpr1-GFP (Fig. 5*F*). In WT, Xpr1-GFP was enriched in the cellular periphery, probably the plasma membrane. In the mutants, Xpr1-GFP was similarly localized to the cell periphery, suggesting that accelerated Pi export in these mutants may not be caused by dynamic alteration of the localization of the exporter, Xpr1.

### Pi hyper-sensitivity of Δpqr1Δxpr1 is suppressed by gene-deletion of Pi transporters Pho84 and Pho842

**Figure 6:**
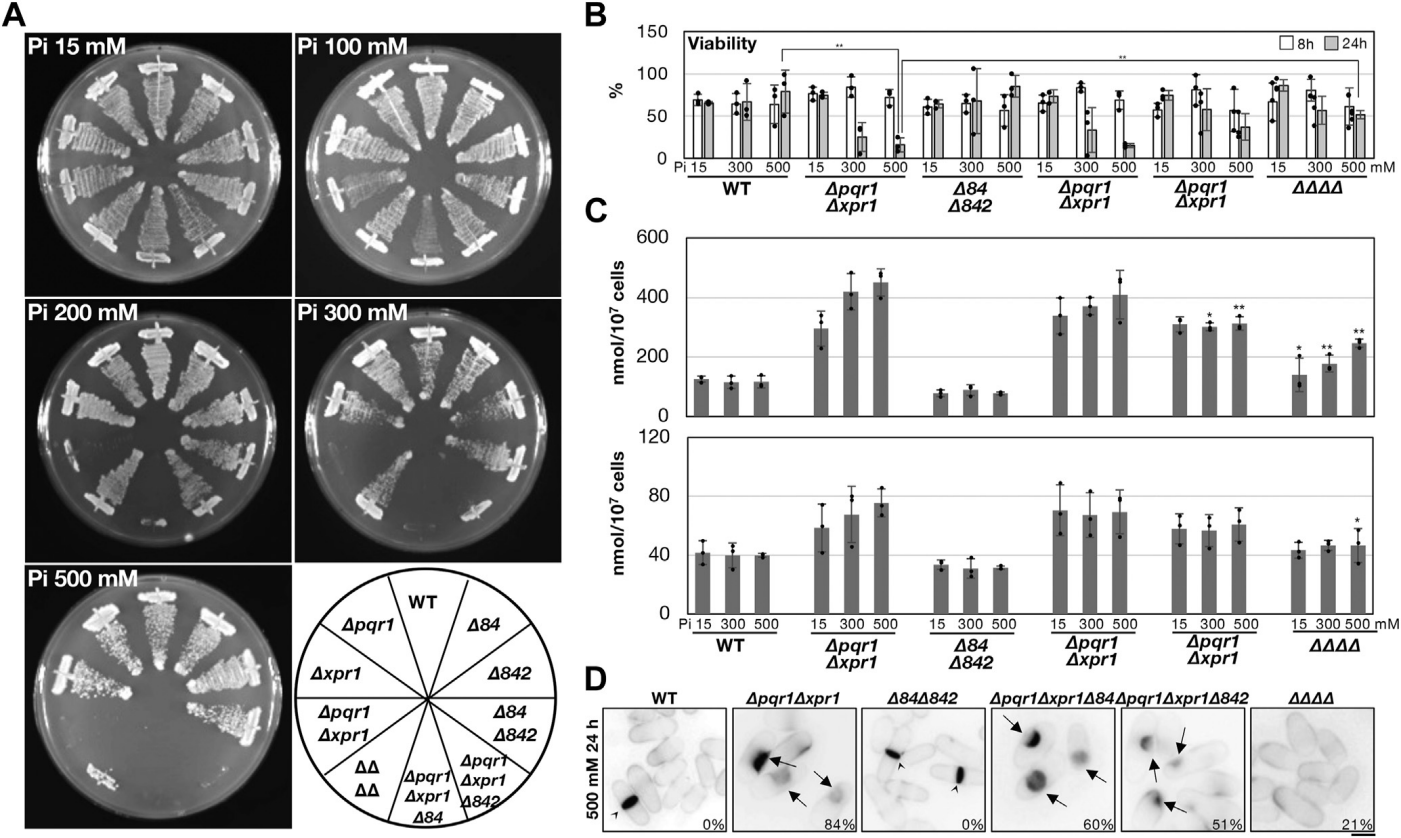
**The Pi hyper-sensitivity of *Δpqr1Δxpr1* was suppressed by the gene-deletion of Pi transporters**. *A*, proliferation of indicated strains on EMM2 plates with various concentrations of Pi (15–500 mM). *Δ84*, *Δ842,* and *ΔΔΔΔ* indicate *Δpho84*, *Δpho842*, and *Δpqr1Δxpr1Δpho84Δpho842*, respectively. *B*, cellular viabilities of indicated strains at 8 and 24 h after medium shift. A double asterisk (∗∗) means the difference is statistically significant (*p* < 0.02). *C*, Pi^total^ and Pi^free^ of indicated strains at 8 h after medium shift. Presented data were normalized by cell numbers (nmol Pi/10^7^ cells). Experiments were repeated three times. Individual data points, means, and SDs were presented. The data of *Δpqr1Δxpr1Δ84*, *Δpqr1Δxpr1Δ842*, and ΔΔΔΔ were analyzed with *Δpqr1Δxpr1*: an asterisk (∗) for significantly different (*p* < 0.05) and a double asterisk (∗∗) for *p* < 0.02. Others were not significantly different from corresponding *Δpqr1Δxpr1* results. *D*, cellular morphologies at 24 h after shift to 500 mM Pi. See Figure 3*E*. Bar represents 5 μm.

Our previous study showed that Pqr1 is required for the internalization of Pho84 and Pho842, the major high-affinity Pi transporters, from the plasma membrane, contributing to the restriction of Pi uptake (36). The double gene deletion of Pho84 and Pho842 suppresses the lethal phenotype of *Δpqr1* in nitrogen-starved conditions. On the other hand, Pqr1 is reportedly involved in transcriptional regulations (47). How does Pqr1 contribute to Pi homeostasis along with Xpr1 and the VTC complex? To obtain insight, we examined the genetic interaction between SPX proteins and Pi transporters (Fig. 6).

The Pi hyper-sensitivity of *Δpqr1Δxpr1* was suppressed by the double gene deletion of Pho84 and Pho842 (Figs. 6*A* and S6). *Δpqr1Δxpr1Δpho84Δpho842* grew as well as WT even at 300 mM Pi and was able to proliferate slightly but reproducibly better than *Δpqr1Δxpr1* at 500 mM Pi, while *Δpqr1Δxpr1* did not form colonies. Both *Δpqr1Δxpr1Δpho84* and *Δpqr1Δxpr1Δpho842* were unable to form colonies at 300 mM Pi, suggesting that Pho84 and Pho842 function redundantly. Consistently, the loss of viability and hyper-accumulation of Pi of *Δpqr1Δxpr1* were significantly suppressed by *Δpho84Δpho842* (Fig. 6, *B* and *C*). *Δpqr1Δxpr1Δpho842* proliferated better than *Δpqr1Δxpr1Δpho84* at 200 mM Pi. The abnormal morphology of *Δpqr1Δxpr1*at higher Pi was also suppressed by *Δpho84Δpho842* (Fig. 6*D*). These results are consistent with the idea that Pqr1 contributes to Pi homeostasis and proliferation through downregulating Pho84 and Pho842. The suppression of the phenotypes of *Δpqr1Δxpr1* by *Δpho84Δpho842* was obvious and significant, but not perfect; *Δpqr1Δxpr1Δpho84Δpho842* formed fewer colonies than WT at 500 mM Pi. Therefore, we do not exclude the following possibilities: remaining Pi transporters, namely Pho841, Pho843, and Plt1, are involved; the other functions of Pqr1, such as transcriptional regulations, reinforce Pi homeostasis achieved through the direct regulation of Pi transporters.

## Discussion

In this study, we investigated functions of Xpr1/Spx2, an SPX-EXS protein and potential Pi exporter, in Pi homeostasis in *S. pombe.* We also investigated the other two SPX factors: Pqr1, a ubiquitin ligase, and Vtc4, an enzymatic subunit of the VTC complex responsible for polyP synthesis. The major outcomes are the following: (1) Xpr1-dependent Pi export activity exists in *S. pombe* and is regulatable, accelerated in the genetic backgrounds of *Δpqr1* and *Δvtc4*; (2) Pqr1, Xpr1, and the VTC complex (the SPX trio) contribute synergistically to Pi homeostasis; (3) Pi homeostasis sustained by the SPX trio is essential for normal vegetative proliferation in the laboratory.

**Figure 7:**
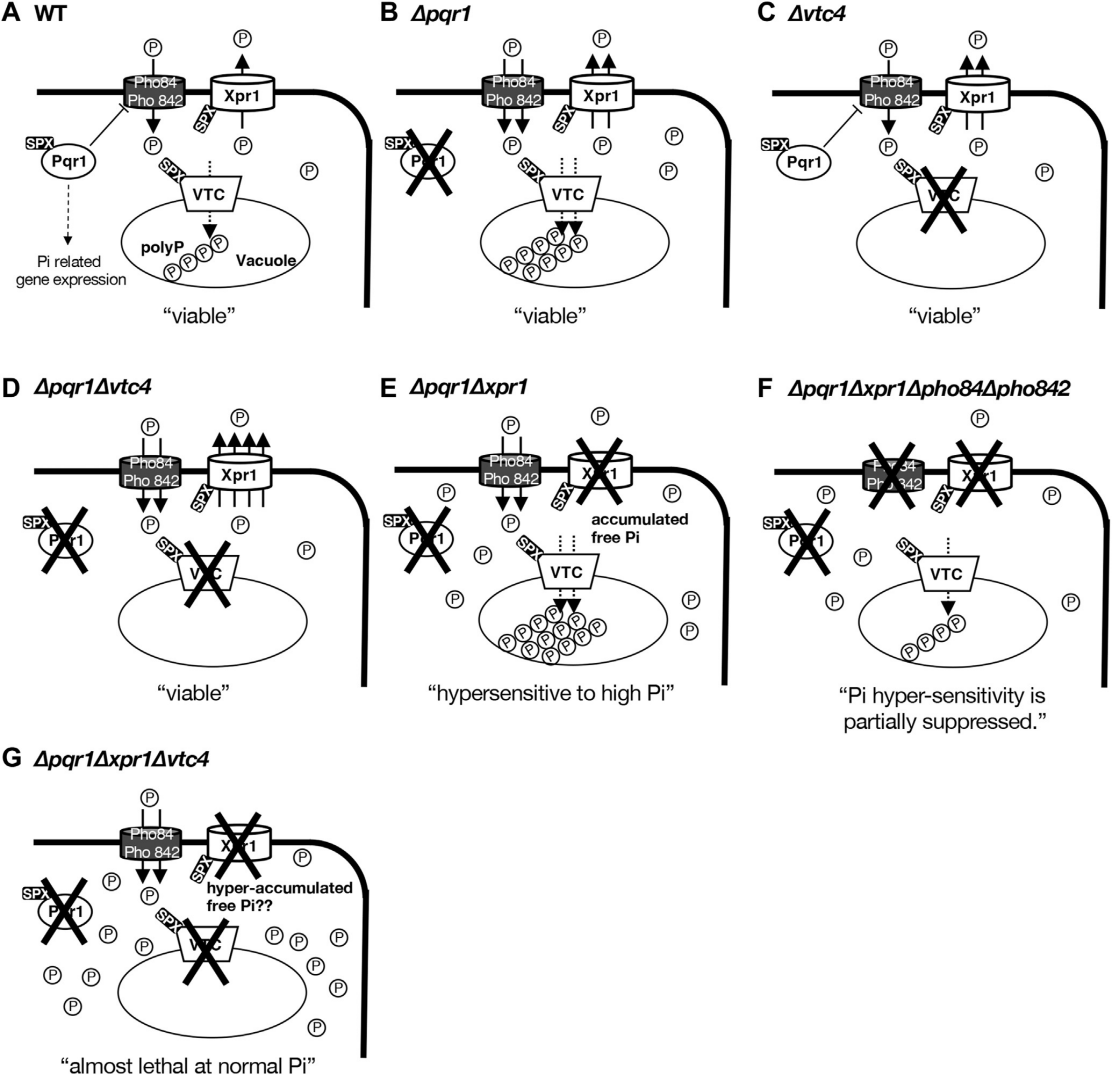
**The working model: The SPX Trio, Pqr1, Xpr1, and the VTC complex contribute synergistically to Pi homeostasis, supporting cell proliferation and viability.***A*, in WT, Pqr1 restricts Pi uptake by downregulating Pho84 and Pho842 Pi transporters (36). Reportedly, Pqr1 may be involved in the regulations of Pi-related genes (47). Xpr1 exports and decreases cellular Pi. The VTC complexes (subunits Vtc2 and Vtc4 contain SPX) on vacuolar membranes synthesize polyP, sequestering a large amount of Pi as polyP in vacuoles. Functions of this trio synergistically sustain Pi homeostasis in the cell. *B*, in *Δpqr1*, the Pi export activity of Xpr1 is accelerated and the VTC complex produces more polyP. *C*, in *Δvtc4*, the Pi export activity of Xpr1 is accelerated. *D*, in *Δpqr1Δvtc4*, the Pi export activity of Xpr1 is accelerated 2-fold more than in *Δpqr1* or *Δvtc4*. *E*, in *Δpqr1Δxpr1*, free Pi accumulates and Pi homeostasis is lost. polyP is assumed to have accumulated but was not examined. *Δpqr1Δxpr1* does not form colonies at high Pi. *F*, hyper-accumulation of cellular Pi and viability loss of *Δpqr1Δxpr1* were suppressed by *Δpho84Δpho842*. *G*, *Δpqr1Δxpr1Δvtc4* forms colonies only at low Pi (∼0.15 mM), probably due to complete loss of Pi homeostasis. polyP, polyphosphate.

First, we propose a working model of how the SPX trio functions in Pi homeostasis (Fig. 7). Xpr1, the SPX-EXS, localizes to the plasma membrane and exports free cytoplasmic Pi to the extracellular environment, reducing [Pi] in the cytoplasm. Pqr1, the SPX-RING ubiquitin ligase, downregulates major Pi transporters, Pho84 and Pho842, and restricts Pi uptake into the cytoplasm (36). Ubiquitination of Pho84 depends mainly on Pqr1 function. The VTC complex, localized to vacuolar membranes, synthesizes polyP in the vacuolar lumen in *S. pombe*, too (36, 46, 47). In budding yeast, it was reported that polyP synthesis by the VTC complex consumes cytoplasmic ATP and probably also occurs in *S. pombe*. Isolating phosphate as vacuolar polyP may result indirectly in the reduction of cytoplasmic Pi. Failure of all three regulatory mechanisms, Pi uptake, Pi export, and polyP synthesis (Pi isolation), disrupts Pi homeostasis, elevating Pi level in the cytoplasm and causing severe growth defects in medium with normal [Pi] (15 mM) or higher (Fig. 2). Regulation by the SPX trio is likely tied to Pi metabolism, as *Δpqr1Δxpr1Δvtc4* is able to proliferate like WT if the Pi concentration is reduced to 0.15 mM (1% of conventional EMM2).

All functions of the SPX trio reduce free [Pi] in the cytoplasm and possibly in the nucleus. In other words, loss of the trio may induce hyper-elevation of free [Pi] in the cytoplasm and/or the nucleus, which cannot be controlled by other cellular systems. Even the double deletion of *pqr1*^*+*^ and *xpr1*^*+*^ caused abnormal elevation of both Pi^total^ and Pi^free^ (Fig. 3). High concentrations of free Pi in the cytoplasm may be lethal, as seen in *Δpqr1Δxpr1*, probably due to widespread disruption of biochemical pathways in the cell. Therefore, we propose a hypothesis: the SPX trio protects the cytoplasm and/or the nucleus by preventing lethal elevation of free Pi. In the cell, Pi is involved in more biochemical reactions than any other compound except water. Hence, uncontrollable elevation of [Pi] interferes with most cellular events. For example, hydrolysis of pyrophosphate, required for transcription and replication, may be affected by hyper-elevated [Pi]. Of note, *S. pombe* inorganic pyrophosphatase Ipp1 is essential for viability (51). Similarly, hydrolysis of ATP is affected. Inefficient hydrolysis of ATP would have a massive impact on all energy metabolism. To evaluate the above hypothesis, it is essential to examine phenotypes of *Δpqr1Δxpr1Δvtc4*, especially free [Pi], although we could not explore this further in the present study, due to the difficulty of handling the triple mutant. Conditional lethal mutants or shut-OFF strains using inducible promoters will be developed to address such questions in the future. Instead, in this study, we thoroughly investigated the second most severe mutant, *Δpqr1Δxpr1*, showing that both Pi^total^ and Pi^free^ were highly elevated and subsequently, viability was lost. As for why elevation of cellular Pi decreases viability of the double mutant, we do not have an experimentally supported hypothesis, although some speculation was already offered above. Morphological abnormalities of *Δpqr1Δxpr1* were readily apparent. Invagination of the plasma membrane was frequently observed. Given that a similar phenotype is invoked by Rho GAP overexpression (49), elevation of cytoplasmic [Pi] might affect Rho functions or cell wall synthesis.

We have provided the first direct evidence of the Pi export activity of Xpr1 in fungi by establishing ^32^Pi export assay using Xpr1-OP strain and gene-deletion mutants of the SPX trio. Furthermore, we have shown that Xpr1-dependent Pi export is vital. Human Xpr1 is medically important for several reasons. It is one of the genes responsible for PFBC. Inhibition of Xpr1 affects serum [Pi] in mice (8, 10, 52). Notably, the zebrafish mutant of Xpr1 lacks microglia, the specialized macrophages in the brain (53). The Pi export activity of Xpr1 has been studied in vertebrates. Regulatory and operating mechanisms for Xpr1 activity are not completely understood, although it is reportedly regulated by binding of PP-IPs to the SPX domain (54). In this study, it was clearly shown that Xpr1-dependent Pi export in *S. pombe* is regulatable and is accelerated in the absence of Pqr1 or Vtc4, without an increase in the Xpr1 protein level or dynamic alteration of its localization. Therefore we speculate that PP-IP–dependent regulation of Xpr1 may be responsible for acceleration of Pi export in *Δpqr1*, *Δvtc4*, and *Δpqr1Δvtc4*, as seen with human Xpr1 (54). Since our understanding of PP-IP metabolism and Pi regulations in *S. pombe* has advanced in recent years through efforts of fission yeast researchers (31, 46, 47, 55), it is interesting to examine Xpr1 activity in mutants of PP-IP metabolic factors, like Asp1, responsible for generation of PP-IP–like IP_8_. In proliferating WT, Xpr1-dependent Pi export was difficult to detect at 15∼500 mM Pi. It is important to explore physiological conditions in which Xpr1 is activated.

While analyzing the results of the ^32^Pi export assay, we realized that a second Pi exporter may exist in *S. pombe*, although Xpr1 has been thought to be the sole Pi exporter in eukaryotes (9). The unidentified Pi exporters may contribute to Pi homeostasis and viability in *Δxpr1* in high Pi. In the future, dosage-suppressor screening should be used to identify such Pi-exporting factors.

Budding yeast Syg1, the potential ortholog of Xpr1, has not been proven to have Pi-exporting activity. If it does, the activity of Syg1 may be undetectable in a WT background, as in *S. pombe*, and it may become detectable only in the mutants of other regulators of Pi homeostasis, for example, *Δpho81* or *Δvtc4* or the double deletion. Syg1 was originally identified as a dosage suppressor for the lethal and cell cycle arrest phenotype of the *gpa1* mutant, a loss-of-function mutant of the α subunit of the heterotrimeric G protein (25). Cellular Pi export activity may be linked to cell cycle machinery.

Pqr1 is an E3 ubiquitin ligase belonging to the SPX-RING family and is orthologous to *NLA* in *A. thaliana* (40) but is not conserved in *S. cerevisiae*. Originally, we identified this ubiquitin ligase as a *proteolysis factor that quiescence requires* (36), while now it is revealed to be important for “*Phosphate Quantity Restriction*.” The Pqr1 substrates are likely major Pi transporters, Pho84 and possibly Pho842, in the plasma membrane. Pqr1 may ubiquitinate Pi transporters and promotes their downregulation by endocytosis, resulting in the restriction of Pi uptake. As shown in the previous study (36), ubiquitination of Pho84 depends on Pqr1 but that of Pho842 is little affected in *Δpqr1* at 15 mM Pi, although internalization of Pho84 and Pho842 are both inhibited in *Δpqr1*. Pqr1 might ubiquitinate a lysine residue of Pho842 important for internalization, and ubiquitination of other lysines of Pho842 might be catalyzed by E3 ligases other than Pqr1. The functional links between Pqr1 and Pi transporters, Pho84 and Pho842, were consolidated with genetic analyses in this study (Fig. 6). Other minor high affinity Pi transporters, Pho841 and Pho843, could be targets of Pqr1, as suppression of Pi hyper-sensitivity of *Δpqr1Δxpr1* by *Δpho84Δpho842* is not perfect. Or Pqr1 could contribute to Pi-related regulations other than downregulating Pi transporters. *NLA* also restricts Pi uptake in *A. thaliana* by downregulation of a Pi transporter (41, 42). It is interesting that *S. cerevisiae* lacks Pqr1.

In Pi homeostasis control in *S. cerevisiae*, the PHO pathway is important because it regulates transcription of the PHO regulon, including Pho5 and Pho84 (12, 13). The core of the PHO pathway is the Cyclin/CDK complex, Pho80/Pho85, and the CDK inhibitor, Pho81, with an SPX domain, controlling a key transcription factor, Pho4 (19). *S. pombe* and many other fungi lack Pho81 but possess an SPX-RING–like Pqr1, instead. The ortholog of Pho85 kinase in *S. pombe* is Pef1, reportedly involved in meiotic regulation and chromosome cohesion, rather than Pi metabolism (33, 34, 35). The core of the PHO pathway is not well conserved in fungi. Therefore, we speculate that Pqr1 may serve additional functions in transcriptional regulation of the PHO regulon.

Recently, Schwer et al. identified mutations of *pqr1*^*+*^/*spx1*^+^ (they call it *spx1*^*+*^; however, to avoid confusion, we use our nomenclature Pqr1 in this manuscript) as extragenic suppressors in an Asp1 pyrophosphatase mutant (47). Asp1 is the key enzyme in PP-IP metabolism and serves dual functions, kinase activity to generate IP_8_ and phosphatase activity for the reverse reaction (46, 55, 56, 57). In the Asp1 pyrophosphatase mutant, excess IP_8_ may cause cytotoxicity that is suppressed by Pqr1 deletion. Therefore, they suggested that Pqr1/Spx1 may act as the transducer of PP-IP signaling for transcriptional regulation of the PHO regulon. Considering that Pqr1 is a ubiquitin ligase, Pqr1 could have dual roles in Pi metabolism, restricting Pi uptake by ubiquitinating Pho84 and mediating PP-IP signaling to regulate the PHO regulon. As PP-IP binds also to the S1 region of budding yeast Pho81 (22), which is distinct from the SPX domain, Pqr1 could have multiple binding sites for PP-IP and could be differentially regulated. It is worth examining whether site-directed mutagenesis on the SPX domain compromises the activity of Pqr1 to restrict Pi uptake. Efforts to find substrates of Pqr1 other than Pi transporters will be also crucial. As Pi regulation is thus varied in species, it is interesting to review Pi regulations of various species in fungi and others at the level of systematic and evolutional biology.

Then, we discuss the VTC complex, the enzymatic subunit of which is Vtc4. The VTC complex, widely conserved in unicellular eukaryotes, but not possessed by metazoans or plants, is a polyP synthase in *S. cerevisiae* (44), as also shown in *S. pombe* by our group and others (36, 46, 47). One of the fundamentally important outcomes of the present study is that the VTC complex is basically essential for proliferation in the genetic background of *Δpqr1Δxpr1*. Given that the VTC complex functions exclusively in polyP synthesis, the above finding may facilitate understanding of essential roles of polyP. We propose the following hypothesis: polyP may be essential to avoid lethal elevation of free Pi in the cytoplasm and/or the nucleus by sequestering Pi as polyP in vacuoles. Our current data are consistent with this hypothesis and we plan to examine this in subsequent studies. As for physiological roles of polyP, many reports have appeared (protein polyphosphorylation (58), protein folding (59, 60), LON protease regulation (61), vacuolar proteolysis required for autophagy (36), transcriptional regulations (62), blood coagulation (63), relevance to amyotrophic lateral sclerosis/ALS (64), replicative lifespan (65), etc. See the following comprehensive reviews (66, 67)). As the amount of polyP in the cell is far greater in yeasts (∼20% of wet weight) than in metazoans and plants, the physiological roles of polyP could vary. However, among nonessential functions of polyP in yeasts, there may be evolutionarily conserved activities, possibly with profound relevance to human health. Despite recent advances of our understanding of metazoan polyP metabolism (68, 69), still many remain elusive. Identification of related enzymes, for example, metazoan polyP synthase, will be key in this research field.

Of the SPX trio: Pqr1, Xpr1/Spx2, and the VTC complex, only Xpr1 is common to all eukaryotes, including humans. Xpr1 is the sole SPX factor in metazoans and may be central to Pi homeostasis in human cells (9, 54). Whereas at the organismal level, Pi metabolism is governed by the endocrine system, individual cells do not need multiple SPX factors to maintain Pi homeostasis. Accordingly, SPX factors other than Xpr1 have been lost evolutionarily, and it is interesting that only Xpr1 remains in metazoans. Understanding the regulatory and operating mechanisms of Xpr1 and its orthologs is in progress; therefore, studies of human Xpr1 may be of broad interest. A syndrome of high [Pi] in serum, hyperphosphatemia, is a risk factor in chronic kidney disease, afflicting over six hundred million people globally (70), and Xpr1 could be a possible therapeutic target for hyperphosphatemia (52). Given that it is also responsible for PFBC, the medical importance of Xpr1 is apparent (8). *S. pombe* is a tractable model organism for genetic and functional studies of human Xpr1 and orthologs.

## Experimental procedures

### Yeast strains, media, and culture

All *S. pombe* strains used are listed in Table S1. Complete and synthetic minimal media, YES (YE with five supplements: adenine, uracil, leucine, histidine, and lysine) and EMM2, were employed (48). To test drug sensitivity, 100 μl of stock solution of each drug (G418, 25 mg/ml; hygromycin B, 75 mg/ml; nourseothricin, 25 mg/ml) were spread on YES or PMG (synthetic medium) plates and yeasts were inoculated. In the case of blasticidin S selection, the drug was spread on YES plate at 0.3 mg/ml (71). PMG contains glutamate as a nitrogen source rather than NH_4_Cl used for EMM2; therefore, it is appropriate for the G418 sensitivity test. For reduced-Pi EMM2 (0.15 mM and 1.5 mM Pi), we first prepared EMM2-P (EMM2 without a Pi source (Na_2_HPO_4_), adjusting the pH to 5.8 with 10 mM MES and NaOH (36)), and normal EMM2 (15 mM Pi) was mixed with EMM2-P. For Pi-increased EMM2 (100 ∼ 500 mM Pi), the appropriate volume of Pi buffer (pH 5.8) was added to EMM2. To prepare solid agar plates of high-Pi media, Pi buffer was added after autoclaving, because the high concentration of Pi interferes with agar gelling. Pi buffer was sterilized using 0.45-μm filters before adding it to the media. Yeast strains were incubated on agar plates or in liquid media at 26 °C. Cellular concentration in liquid media was measured with a CDA-1000 particle counter (Sysmex).

### Yeast genetics

Gene deletion and epitope tagging were performed as described previously (61). To generate double or triple gene-deletion mutants, random spore analysis or tetrad analysis was performed.

### Bright-field and fluorescent microscopy

Cell images were acquired using an Axiovert 200M fluorescence microscope (Carl Zeiss) or a confocal microscope setting LSM800 (Carl Zeiss). CW (Fluorescent Brightener 28; Sigma-Aldrich) was used to stain cell walls after fixing cells with glutaraldehyde. For observation of GFP fluorescence, Xpr1-GFP–expressing cells were cultured in EMM2 at 26 °C to mid-log phase and were not fixed before microscopy. The Axiovert 200M was used for Figure 1, Figure 2, Figure 3 and 6 and the LSM800 for Figure 5.

### Protein extraction and immunoblot analysis

To extract Xpr1-GFP, harvested cells were suspended in ice-cold TEG buffer (20 mM Tris–HCl (pH 8.0), 1 mM EDTA, 10% (v/v) glycerol, 150 mM NaCl, 1% (v/v) Triton-X100, 1 mM PMSF, and a protease inhibitor cocktail (Roche)) and were crushed with glass beads at 0 °C using a Multi-beads shocker (Yasui Kikai). Cell extracts were centrifuged at 5000 rpm for 5 min and supernatants were incubated on ice for 120 min in the presence of LDS-PAGE sample buffer and 2-mercaptoethanol. For efficient extraction of membrane proteins, like Xpr1, incubation at a temperature lower than boiling is important. Identical amounts of protein (around 30 μg/lane) were separated on SDS-PAGE gels and transferred to nitrocellulose membranes. Anti-GFP (1/1000, Roche) and anti-PSTAIRE (1/1000, SIGMA) Cdc2 monoclonal antibodies were used as primary antibodies. Skim milk (2%) in PBS was used for membrane blocking and antibody dilution. Horseradish peroxidase–conjugated secondary antibodies (Promega) and Clarity Western ECL substrate (Bio-Rad) were used. Chemiluminescent signals were detected with a Lumino-Image Analyzer LAS 4000 (GE Healthcare) and were analyzed with ImageJ (https://fiji.sc) software.

### Quantification of cellular total Pi and free Pi

To quantify intracellular total Pi, Pi^total^, we followed the procedure of Sawada *et al*. (36). In short, harvested cells (5 × 10^7^ cells) were suspended in 200 μl of 1 M H_2_SO_4_ and were boiled at 95 °C for 30 min to extract intracellular Pi. After boiling, the extract was neutralized with 1N NaOH. [Pi] was measured with a Malachite Green Phosphate Assay Kit (R&D Systems, Inc.). As for intracellular free Pi quantification, Pi^free^, cell extracts were directly employed in the Malachite Green assay. Harvested cells (0.5 ∼ 1 × 10^8^ cells) were suspended in 400 μl of extraction buffer (10 mM Tris–Cl (pH 8.0), 1 mM EDTA, 100 mM NaCl, 0.1% (v/v) Triton X-100). Then 300 μl of TE-saturated phenol (FUJIFILM) and appropriate quantities of glass beads were added to the cell suspension in 2-mL screw-capped tubes. Cells were disrupted with a Multi-Beads shocker (Yasui Kikai) at 0 °C. To cell extracts recovered from glass beads, 300 μl of chloroform were added and tubes were vortexed and centrifuged for 5 min at 14,000 rpm. The separated water phase was transferred to a new tube, followed by the addition of 300 μl of chloroform. Then, tubes were vortexed and centrifuged. Separated water phases were transferred to new tubes and frozen in liquid N_2_. Extract aliquots were appropriately diluted with water and the concentration of Pi was measured with a Malachite Green Phosphate Assay Kit.

### Pi export assay

*S. pombe* cells were cultured in normal EMM2 at 26 °C to mid-log phase (∼5 × 10^6^ cells/ml), harvested with centrifugation, and washed twice with EMM2-P. For overproduction of Xpr1-GFP with *nmt41* promoter, cells were cultured in normal EMM2 without thiamin for 18∼19 h. Radioactive monopotassium phosphate solution (KH_2_^32^PO_4_, 74 MBq/ml, NEX060, PerkinElmer) was diluted 10-fold with EMM2-P, and 50 μl of the diluted solution was added to 450 μl of cell suspension containing ∼5 × 10^7^ cells and incubated at 26 °C for 30 min for ^32^PO_4_ uptake. To measure cellular concentration, an automated cell counter Countess II (Thermo Fisher Scientific) was used. Then, cells were washed three times with EMM2-P, and cell pellets were suspended in 850 μl of EMM2 containing 15 or 500 mM Pi. At indicated time point, 200 μl of the suspension was withdrawn, centrifuged twice for 1 min at 13,000 rpm to remove cells, and an aliquot (50 μl) of the supernatant was added to a scintillator (10 ml in a vial), Ecoscent ultra (National Diagnostics). Radioactivity was measured in a liquid scintillation counter AccuFLEX LSC-8000 (Hitachi). Radioactivities of cell suspensions at 0 min were also measured to determine amounts of total incorporated ^32^P. In Figure 4, *C*–*F*, radioactivities of the supernatants at 0 min were subtracted from those at 0, 20, 40, and 60 min, and ratios of those values to total amounts of incorporated ^32^P were calculated as percentages. All experiments were performed in triplicate.

## Data availability

All the data described are located in this article.

## Supporting information

This article contains supporting information (36).

## Conflicts of interest

The authors declare that they have no conflicts of interest with the contents of this article.
