# Supplementary material for: Phosphate uptake restriction, phosphate export, and polyphosphate synthesis contribute synergistically to cellular proliferation and survival
Source: J Biol Chem. 2023 Nov 8;299(12):105454. doi: 10.1016/j.jbc.2023.105454 (PMC10704438; doi:10.1016/j.jbc.2023.105454)
Supplement: Supporting information [file mmc1.docx]

**Supportive Information**

**Phosphate uptake restriction, phosphate export, and polyphosphate synthesis contribute synergistically to cellular proliferation and survival**

Masahiro Takado^3^, Tochi Komamura^1^, Tomoki Nishimura^1^, Ikkei Ohkubo^1^, Keita Ohuchi^1^,

Tomohiro Matsumoto^3^ and Kojiro Takeda^1,2,#^

^1^Graduate School of natural science, ^2^Institute of Integrative neurobiology, Konan University

^3^Radiation Biology Center, Graduate school of Biostudies, Kyoto University

**The List of Supportive Information:**

Table S1. The list of *S. pombe* strains

Figure S1. Pi^total^ comparison.

Figure S2. Pi concentration in YES complete medium.

Figure S3. Cell morphologies on agar plates.

Figure S4. Cell morphologies of WT, *∆pqr1*, *∆xpr1,* and *∆pqr1∆xpr1*.

Figure S5. Expression check of Xpr1-GFP overproduction.

Figure S6. Growth curve of *∆pqr1∆xpr1∆pho84∆pho842* at various [Pi].

**Table S1 The list of *S. pombe* strains used in this study**

| **Strain** | **Genotype** | **Source** |
| --- | --- | --- |
| 972 | WT h^-^ |  |
| KP573 | h^-^ *∆pqr1*::hphMX | Sawada et al.^36^ |
| SN178 | h^-^ *∆vtc4*::kanMX | Sawada et al.^36^ |
| TKN46 | h^-^ *∆xpr1*::kanMX | BIONEER derived |
| TKN51 | h^-^ *∆xpr1*::kanMX *∆vtc4*::natMX | This study |
| TKN55 | h^-^ *∆pqr1*::hphMX *∆xpr1*::kanMX | This study |
| TKN97 | h^-^ Xpr1-GFP::KanMX | This study |
| TKN98 | h^+^ *∆pqr1*::hphMX Xpr1-GFP::KanMX | This study |
| TKN99 | h^+^ *∆vtc4*::bsd^*^ Xpr1-GFP::KanMX | This study |
| TKN100 | h^-^ *∆pqr1*::hphMX *∆vtc4*::bsd Xpr1-GFP::KanMX | This study |
| TsK33 | h^-^ *∆pqr1*::hphMX *∆xpr1*::kanMX *∆vtc4*::natMX | This study |
| SN180 | h^+^ *∆pho84*::natMX | This study |
| SN127 | h^-^ *∆pho842*::kanMX | This study |
| SN230 | h^-^ *∆pho84*::natMX *∆pho842*::kanMX | Sawada et al.^36^ |
| KmT8 | h^-^ *∆pqr1*::hphMX *∆xpr1*::bsd *∆pho84*::natMX | This study |
| KmT10 | h^-^ *∆pqr1*::hphMX *∆xpr1*::bsd *∆pho842*::kanMX | This study^$^ |
| KmT14 | h^-^ *∆pqr1*::hphMX *∆xpr1*::bsd *∆pho84*::natMX *∆pho842*::kanMX | This study^$^ |

*: Blasticidin S resistant gene


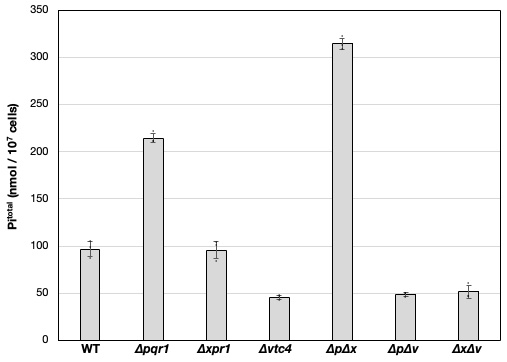


**Figure S1. Pi^total^ comparison.**

Pi^total^ in single gene-deletion mutants and double gene-deletion mutants of *pqr1^+^*, *xpr1^+,^* and *vtc4^+^*. Data for strains other than *∆xpr1∆vtc4* were the same as shown in Fig. 1A. Experiments were repeated 3x, and individual data points, means and SDs are presented.

**
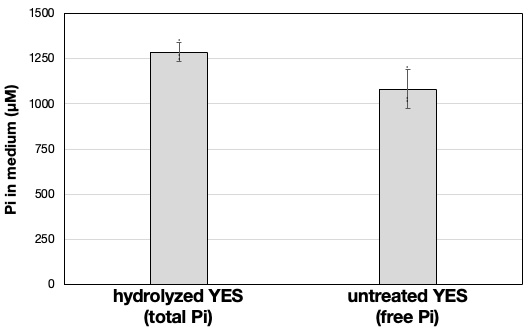
**

**Figure S2. Pi concentration in YES complete medium.**

YES, a liquid medium was hydrolyzed in 1 M H_2_SO_4_ for 30 min at 100 °C. The solution was neutralized with 1 M NaOH and the Pi concentration was measured using the malachite green method (hydrolyzed YES/total Pi). Alternatively, YES medium was diluted with water and the Pi concentration was measured directly (untreated YES/free Pi). In either case, YES contains approximately 1 mM Pi. Experiments were repeated 3x, and individual data points, means and SDs are presented.


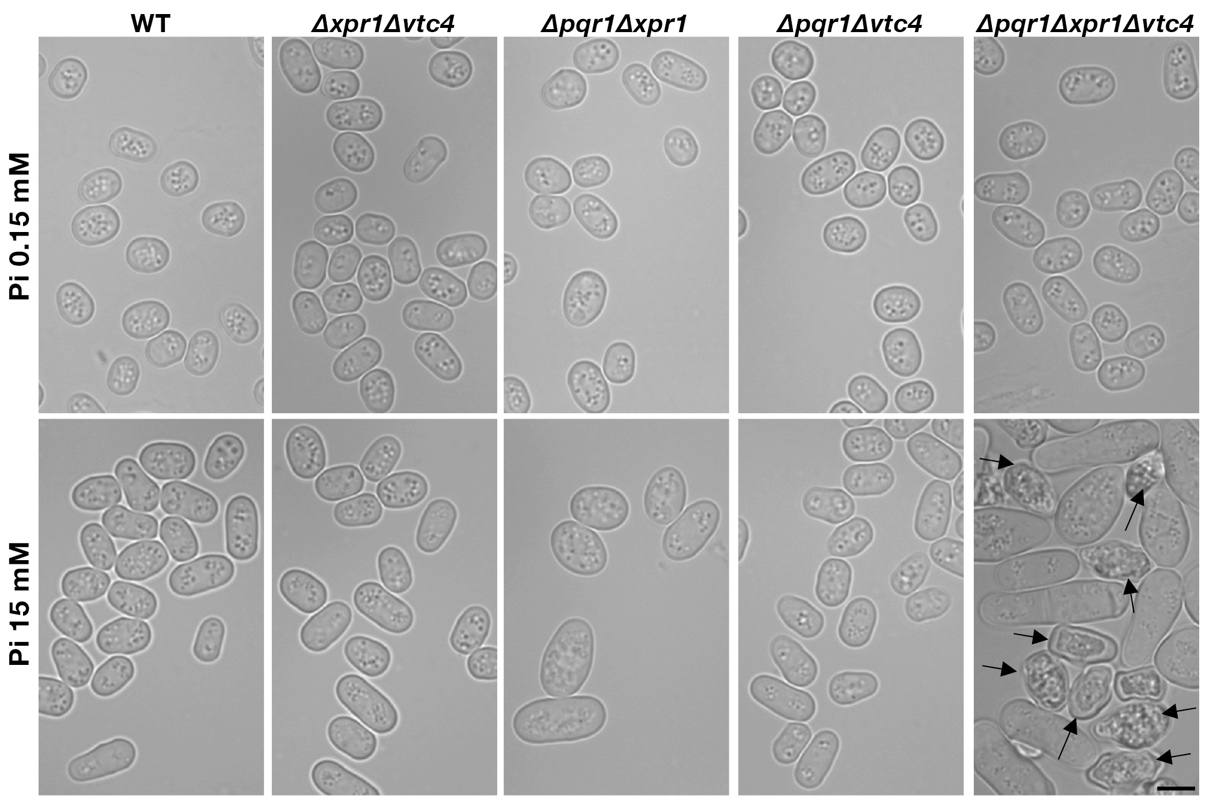


**Figure S3. Cell morphologies on agar plates.**

Cells grown on plates were observed with conventional microscopy. *∆pqr1∆xpr1∆vtc4* cells showed abnormal morphologies and many were collapsed on the 15 mM Pi plate (arrows), but no such abnormalities were observed on 0.15 mM Pi plates. Other strains showed no collapsed cells on either 0.15 mM or 15 mM Pi plate. The images of WT and *∆pqr1∆xpr1∆vtc4* (0.15 mM and 15 mM) are the same as those shown in Fig. 2D. Bar = 5 µm.

**
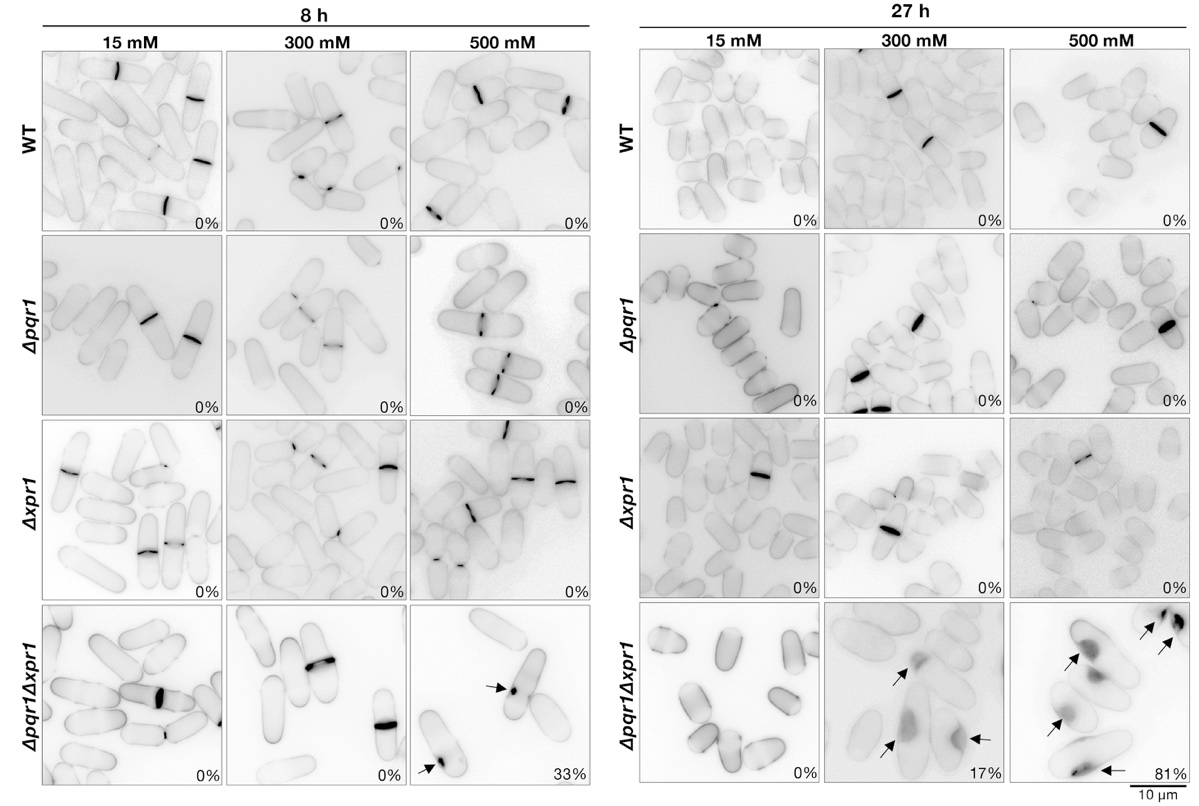
**

**Figure S4. Cell morphologies of WT, *∆pqr1*, *∆xpr1,* and *∆pqr1∆xpr1*.**

Cellular morphologies were examined with Calcofluor White (CW) staining of cell walls. Cells of indicated strains were fixed with glutaraldehyde and stained with CW, 8 and 27 h after shifting to 15, 300, and 500 mM Pi EMM2. Structures indicated by arrowheads are septa formed before cytokinesis. Arrows indicate abnormal invaginations observed in *∆pqr1∆xpr1*. Materials stained with CW were deposited in the invaginated space. The percentage of cells with invagination is shown. The images of WT (8 h 15 mM and 500 mM), WT (27 h 15 mM and 500 mM), ∆pqr1∆xpr1 (8 h 15 mM and 500 mM), and ∆pqr1∆xpr1 (27 h 15 mM and 500 mM) are the same as those shown in Fig. 3E. Bar = 10 µm.


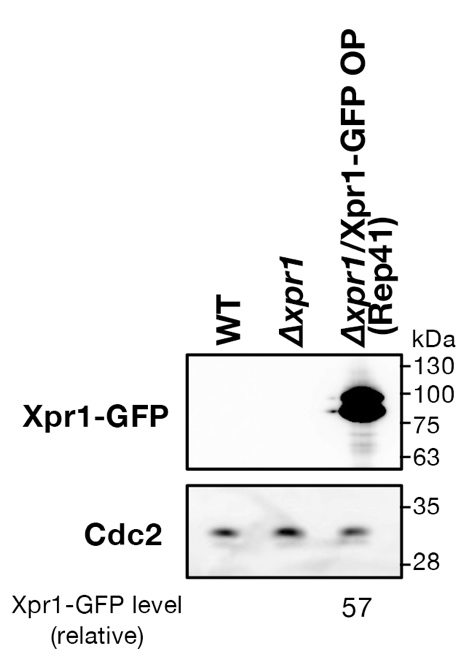


**Figure S5. Expression check of Xpr1-GFP overproduction**

The expression of Xpr1-GFP of strains used in Fig. 4 was confirmed by anti-GFP immunoblot. Xpr1-GFP expression, controlled by the *nmt41* promoter, was induced for ~18h by withdrawing vitamin B1 from the media. As for quantification, see the legend of Fig. 5 and materials & methods.


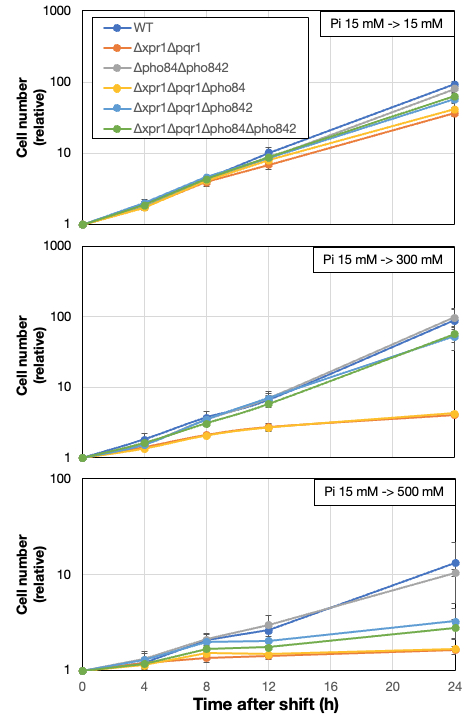


**Figure S6. Growth curve of *∆pqr1∆xpr1∆pho84∆pho842* at various [Pi]**

The indicated strains were cultured at 15 mM Pi and then shifted to 15 (top), 300 (middle), or 500 (bottom) mM Pi EMM2. Y-axes indicate cell concentrations in the media measured with a particle counter and relative values are plotted (setting value at 0 h to 1.0). Experiments were repeated three times. Means and SDs were presented.
